# Age-specific relationship between the modulation of brain dynamics in response to task demands and bimanual performance

**DOI:** 10.18632/aging.206363

**Published:** 2026-03-24

**Authors:** Sara Magalhães Ferreira, Maud Beeckmans, Joana Frieske, Raf Meesen, Stephan P. Swinnen, Koen Cuypers

**Affiliations:** 1 Neuroplasticity and Movement Control Research Group, Rehabilitation Research Institute (REVAL), Hasselt University, Diepenbeek, 3590 Limburg, Belgium; 2 Movement Control & Neuroplasticity Research Group, Department of Movement Sciences, Group Biomedical Sciences, KU Leuven, Heverlee, 3001 Flemish Brabant, Belgium; 3 Leuven Brain Institute (LBI), KU Leuven, Leuven, 3000 Flemish Brabant, Belgium; 4 Medical Imaging Research Center, UZ Leuven, Leuven, 3000 Flemish Brabant, Belgium

**Keywords:** aging, bimanual coordination, Bimanual Tracking Task, BOLD variability, task modulation

## Abstract

While prior research has largely focused on mean Blood Oxygen Level-Dependent (BOLD) activation to understand age-related differences in bimanual coordination, BOLD variability - a metric that captures fluctuations in brain activity -, has been overlooked. Hence, the current study examined how age affects BOLD variability, specifically BOLD standard deviation (BOLD SD), and its modulation with task demands during a bimanual task. Twenty-two younger and twenty-three older adults performed three task conditions of increasing complexity while undergoing functional magnetic resonance imaging (fMRI).

Older adults exhibited higher BOLD SD in cerebellar lobule VIIIb and greater modulation across task conditions in both sensorimotor and cerebellar regions. Modulation of BOLD variability predicted task performance in an age- and region-dependent manner: in younger adults, reduced modulation in sensorimotor and visuospatial areas correlated with better performance, whereas in older adults, increased modulation in the inferior and superior parietal lobules was linked to higher performance. Across groups, better outcomes were predicted by greater modulation in the middle occipital gyrus but less in the cerebellar Crus I. These findings underscore an age-related shift in the neural dynamics underpinning motor adaptability with aging, pointing to increased BOLD variability modulation as a potential marker of compensatory reorganization in late adulthood.

## INTRODUCTION

Preserving the ability to coordinate both hands efficiently in space and time is crucial for executing a vast repertoire of daily activities (e.g., getting dressed, driving a car, and eating with a fork and knife) that are essential for maintaining autonomy and functional independence [1, 2]. However, increasing age is characterized by impaired bimanual coordination manifested by increased task variability [3] and reduced capacity to inhibit between-hand interference, particularly during complex bimanual movements with asymmetric hand-timing demands which require greater executive control [4, 5]. These deficits have been linked to alterations in white matter integrity [3] and diminished differentiation of functional systems [6]. Nonetheless, older adults seem to retain their capacity to modulate brain activity according to task complexity by recruiting additional higher-order cortical areas relative to younger adults to support bimanual performance [6–8]. This suggests that age-related differences in bimanual motor control likely reflect a complex interplay between neural decline and compensatory adaptation that existing cognitive aging theories such as the Dedifferentiation Theory [9], the Scaffolding Theory of Aging and Cognition (STAC) [10] or the Compensation-Related Utilization of Neural Circuits Hypothesis (CRUNCH) [11] account for to varying degrees.

While functional magnetic resonance imaging (fMRI) has been extensively used to address age-related brain activity differences underlying bimanual performance, conventional analyses focus on blood-oxygen-level-dependent (BOLD) signal activation, neglecting the unique, informative potential of BOLD signal variability. Unlike static, mean-based measures, BOLD variability metrics such as BOLD SD (i.e., the standard deviation of the BOLD time series across voxels) capture the moment-to-moment fluctuations in BOLD signal intensity, providing a more comprehensive picture of the dynamic adaptability of neural systems [12]. This emerging fMRI metric was found to be an age-sensitive source of information orthogonal to mean BOLD and is considered to reflect neural efficiency and adaptability to the ever-changing internal and external demands [13–18]. Even though the exact physiology behind BOLD variability remains unclear, it likely reflects a combination of synaptic excitatory-inhibitory balance [19, 20] and interactions between vascular and neuronal factors [21, 22].

In past studies, BOLD variability has been described to change across the lifespan in an inverted U-shape pattern peaking around young adulthood [14, 15], with greater variability generally associated with better cognitive performance [14, 15, 23, 24] as corroborated by recent work from Steinberg and King [25]. By compiling evidence on BOLD variability across a wide variety of cognitive functions, Steinberg and King [25] concluded that, in general, greater BOLD variability benefits task performance, resulting in improved accuracy and lower reaction times. These results are congruent with the Stochastic Resonance Theory, which postulates that neural noise can facilitate processing efficiency by increasing the sensitivity of neural systems to incoming information [26].

Contrasting the findings from Garrett et al. [14, 15], Boylan et al. [27] indicate that the relationship between BOLD variability and age is region-dependent, with older adults typically displaying reduced BOLD variability in cortical regions, relative to younger adults, but increased in subcortical regions. These alterations seen subcortically are potentially driven by reduced dopaminergic neuromodulation [28, 29] or age-related disruptions in structural integrity [30]. Notably, other studies have linked higher BOLD variability in older adults to white matter hyperintensities (WMH), poorer cognition, and reduced dopamine D1 receptor binding [28, 31, 32], suggesting a potential detrimental role of higher BOLD variability in old adulthood.

Research on age-related differences in the modulation of BOLD variability - that is, how BOLD variability changes in response to varying task complexity -, is equally dissonant. Even though higher intra-individual variability across different experimental conditions was generally reported in younger adults relative to older adults [25, 33, 34] the direction of the regulation (i.e., up or down) varied according to the study in question. For instance, whereas results from Boylan et al. [27] pointed out a negative relationship between BOLD variability and task difficulty in younger adults but a positive association in older adults, Rieck et al. [34] reported downregulation across the lifespan. Other studies found instead a consistent up-modulation in both younger and older adults [25, 27, 35, 36]. These discrepancies in findings may stem from differences in the brain regions analyzed, the specific tasks or task conditions used, or even in the experimental design and the BOLD variability metric computed (see [18] for a discussion on the impact of these factors). Therefore, it remains uncertain which direction of modulation (i.e., up- or downregulation) with task demands is detrimental or beneficial in aging. Nonetheless, altered modulation patterns in older adults (relative to younger adults) have frequently been associated with slower, less accurate and less stable behavioral performances in- and out-of-scanner [27, 33, 34, 36].

Despite the growing number of studies investigating BOLD variability in the context of healthy aging, up-to-now, the focus still lies on cognitive functions. To our knowledge, no study has yet investigated how age-related changes in BOLD variability – and in its modulation with task difficulty - affect complex motor functions such as bimanual coordination, despite its immensurable potential. Given that BOLD variability is considered to carry meaningful information beyond mean signal and has been shown to predict age with greater accuracy than traditional activation measures [14], it may serve as a sensitive marker for detecting subtle neural changes that underlie age-related motor impairments, and inform about novel interventions for preserving motor abilities across the lifespan. Moreover, considering the cognitive demands and executive control requirements of complex bimanual tasks [4], it is likely that BOLD variability also modulates motor performance, similarly to how it does in cognitive domains. Hence, examining BOLD variability and its task-dependent modulation during sensorimotor tasks could not only extend our understanding of the neural mechanisms of aging but also provide empirical evidence supporting or contesting current theoretical models of cognitive aging.

To address this gap, the present study examined BOLD variability in younger and older adults during a bimanual coordination task. Specifically, we sought to: 1) examine age-related differences in BOLD variability and its modulation across distinct task demands, and 2) explore how these age differences in the neural dynamics relate to bimanual performance. By doing so, this work sought to elucidate how intrinsic fluctuations in brain activity contribute to motor adaptability and bimanual coordination in aging, potentially offering new insights into mechanisms of functional decline and compensation.

In line with the Stochastic Resonance Theory [26] and recent findings from the review by Steinberg and King [25] suggesting that higher signal variability reflects greater neural flexibility to switch between brain states efficiently [16], we hypothesized that older adults would exhibit less overall BOLD variability during the task and reduced upregulation in response to increasing task difficulty relative to younger adults. Furthermore, we predicted that these differences in brain activity would be associated with relatively poorer bimanual performance in the older group, reflecting diminished dynamic adaptability in sensorimotor control systems.

## RESULTS

### Behavioral data

### Effect of age group and task condition on BTT performance

**Figure 1:**
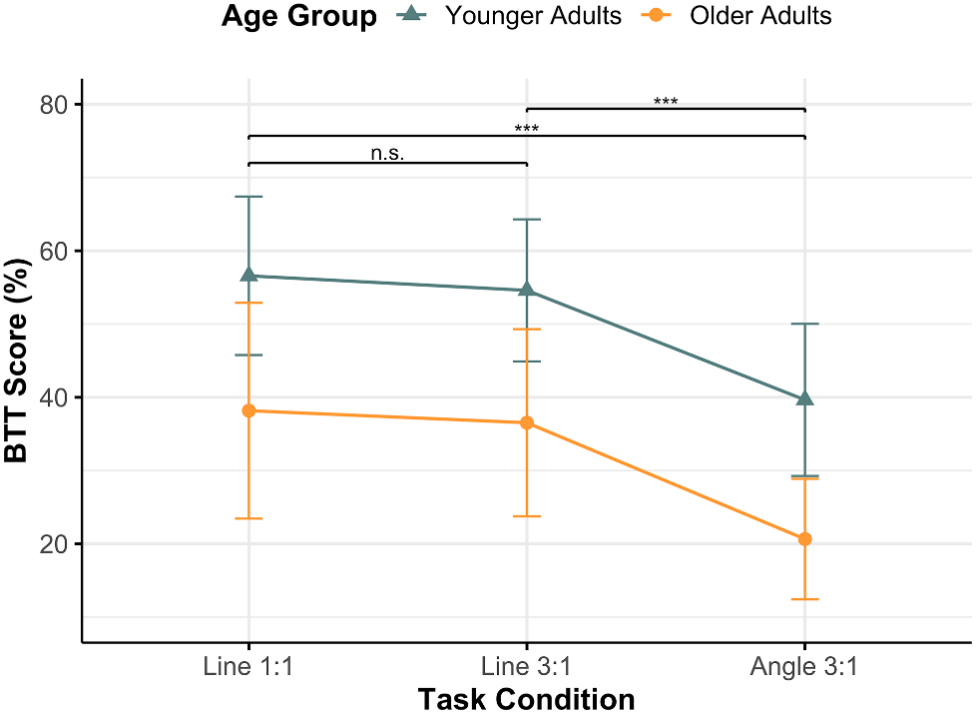
**Mean BTT scores across task conditions per age group.** The figure shows mean BTT scores (%) for each task condition (Line 1:1, Line 3:1, and Angle 3:1) in Younger Adults (teal, triangle) and Older Adults (orange, circle). Whiskers indicate standard deviation. Both groups achieved their highest scores in Line 1:1 and their lowest in Angle 3:1. Following a significant main effect of TASK CONDITION, post hoc Tukey Honestly Significant Difference tests revealed that Angle 3:1 differed significantly from both Line 1:1 and Line 3:1 (*** *p* < 0.001), while the two “Line” conditions did not differ (n.s.). Abbreviations: BTT = Bimanual Tracking Task; n.s. = non-significant.

A 2 x 3 Mixed Model ANOVA was performed to evaluate the effects of AGE GROUP and TASK CONDITION on BTT SCORE. The means and standard deviations for BTT SCORE are presented in Table 1 and can be visualized in Figure 1.

**Table 1:** Descriptive statistics for BTT score.

**Task condition**	**Younger adults**			**Older adults**			**Overall**	
	***Mean***	***SD***		***Mean***	***SD***		***Mean***	***SD***
Line 1:1	56.58	10.81		38.17	14.73		47.17	15.84
Line 3:1	54.60	9.69		36.53	12.77		46.20	14.91
Angle 3:1	39.65	10.38		20.65	8.23		29.94	13.32
Overall	50.27	12.69		31.78	14.43		40.82	16.43

Mean and standard deviation (*SD*) values for BTT score across different age groups (Younger Adults and Older Adults) and task conditions (Line 1:1, Line 3:1 and Angle 3:1).

The two-way ANOVA revealed significant main effects of AGE GROUP (F[1,129] = 29.71, *p* < 0.001) and TASK CONDITION (F[2,129] = 16.80, *p* < 0.001), indicating that BTT performance differed between younger and older adults and varied across task conditions. As expected, younger adults (mean ± SD = 50.27 ± 12.69) performed on average better than older adults (mean ± SD = 31.78 ± 14.43). Moreover, participants overall performed worse on the Angle 3:1 (mean ± SD = 29.94 ± 13.32) comparatively to Line 3:1 (mean ± SD = 46.20 ± 14.91) and Line 1:1 (mean ± SD = 47.17 ± 15.84). Post-hoc Tukey HSD comparisons confirmed that Angle 3:1 differed from “Line” conditions (*q* < 0.001), whereas Line 1:1 and Line 3:1 did not differ from one another (*q* = 0.826). Lastly, the AGE GROUP × TASK CONDITION interaction was not significant (F[2,129] = 0.019, p = 0.981), suggesting that the effect of task condition on BTT performance did not differ between age groups.

### Neuroimaging data

### Effect of age group and task condition on framewise displacement

We examined differences in framewise displacement (FD) across age groups and task conditions using an Aligned Rank Transform (ART) factorial ANOVA to assess the potential confounding effect of head motion on BOLD SD estimates.

**Figure 2:**
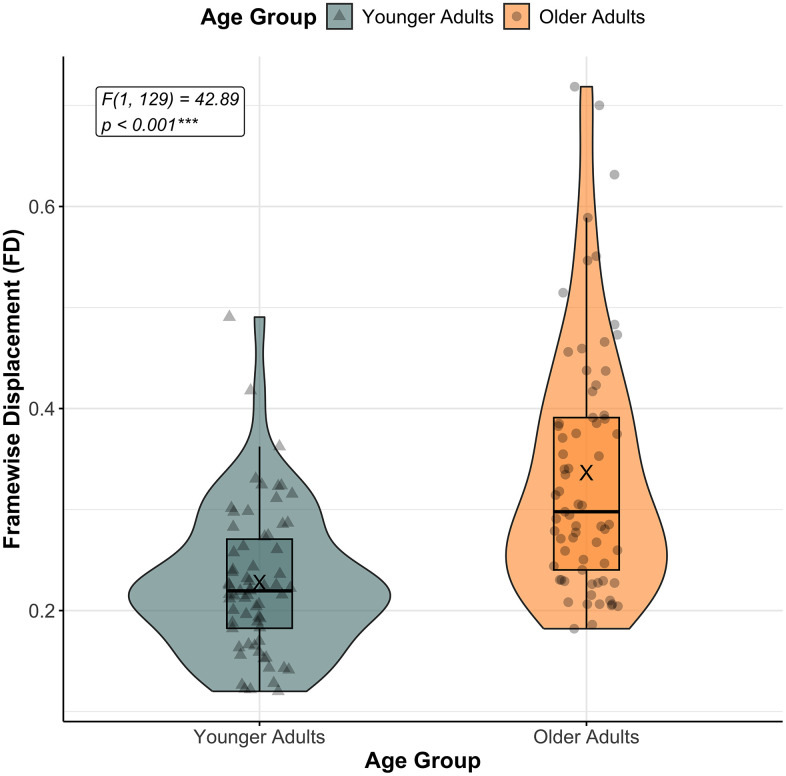
**Framewise displacement (FD) across age groups.** The figure shows framewise displacement (FD) across age groups (Younger Adults in teal, and Older Adults in orange) while performing the Bimanual Tracking Task (BTT). The “X” indicates mean FD for each age group. A significant main effect of AGE GROUP was observed (the corresponding *F*-value and *p*-value are reported), with older adults presenting overall more framewise displacement compared to younger adults. Individual data points reflect mean FD per subject, pooled across task conditions. Significance level: *** *p* < 0.001.

A significant main effect of AGE GROUP was found (F[1,129] = 42.89, *p* < 0.001), indicating that older adults (mean ± SD = 0.34 ± 0.12) exhibited greater FD during BTT performance compared to younger adults (mean ± SD = 0.23 ± 0.07). However, there was no significant main effect of TASK CONDITION (F[2,129] = 0.39, *p* = 0.675), suggesting that movement-related artifacts did not systematically differ across task conditions. Additionally, the AGE GROUP × TASK CONDITION interaction was not significant (F[2,129] = 0.17, *p* = 0.843), indicating that the relationship between BTT conditions and FD did not differ across age groups. These results are depicted in Figure 2.

These findings indicated a potential bias in BOLD SD estimation in older adults compared to younger due to more head motion, reinforcing the importance of controlling for FD in subsequent analyses.

### Age-group differences in BOLD variability and BOLD variability modulation during BTT performance

#### Effect of age group and task condition on BOLD variability

To investigate whether BOLD variability differed across age groups and task conditions in the selected brain regions, a LMM was conducted per ROI, controlling for FD. Subject ID was included as a random intercept to account for within-subject variance. Box-Cox transformations were applied in 21 ROIs to meet model assumptions (see Table 2 for the λ values used in the transformation). On average, two data points of BOLD SD were removed per ROI (i.e., 1.5 % of the total data points; the range of data points excluded was from zero to four), as they were more than three standard deviations of the ROI’s overall mean BOLD SD.

**Table 2:** Summary of age group, task condition and interaction effects on BOLD variability across ROIs.

**ROIs**		**λ**		**Age group**			**Task condition**			**Age group x task condition**	
				***F***	***q***		***F***	***q***		***F***	***q***
RH	A2	0.125		6.27	**0.129**		-	-		-	-
LH	A37vl	-0.625		-	-		-	-		-	-
RH	A39c	-		-	-		-	-		-	-
LH	A40rd	-0.654		-	-		-	-		-	-
RH	A5l	-		-	-		-	-		-	-
LH	A6cdl	-0.033		0.87	0.986		1.63	0.628		3.11	0.279
LH	A6dl	-0.801		-	-		3.41	**0.313**		-	-
RH	A6dl	0.130		5.15	**0.172**		-	-		-	-
LH	A6vl	-0.431		-	-		-	-		-	-
RH	A7c	-1.634		-	-		-	-		-	-
RH	A7ip	-0.255		-	-		-	-		-	-
RH	A7m	-0.092		3.45	0.287		1.18	0.426		3.15	**0.279**
LH	A7r	-		-	-		4.43	**0.278**		-	-
LH	iOccG	-0.814		-	-		-	-		-	-
LH	IsOccG	-3.000		5.07	**0.172**		4.74	**0.278**		-	-
LH	mOccG	-0.860		8.12	**0.079**		-	-		-	-
LH	OPC	-0.547		-	-		-	-		-	-
LH	V5/MT+	-0.800		0.64	1.000		3.07	0.323		4.17	**0.279**
RH	V5/MT+	-1.043		0.11	1.000		2.47	0.375		3.52	**0.279**
LH	Crus I	0.206		1.27	0.829		0.18	1.000		3.97	**0.279**
RH	Crus I	-1.788		2.14	0.536		0.07	1.000		4.73	**0.279**
LH	VI	-		-	-		-	-		-	-
RH	VI	-1.017		0.11	1.000		2.76	0.345		4.00	**0.279**
LH	VIIb	0.145		-	-		-	-		-	-
LH	VIIIb	-0.330		13.52	0.014^*^		-	-		-	-

Summary of results from the Linear Mixed Model (LMM) conducted per ROI to investigate whether BOLD variability differed across age groups and task conditions while controlling for framewise displacement (at the significance level of *q* < 0.05, after False Discovery Rate [FDR] correction for multiple comparisons). Values in **bold** reflect significant effects before FDR correction (*p* < 0.05). A dash (“-”) indicates that the corresponding term was excluded from the final model following stepwise simplification, or that no Box-Cox transformation was applied to the data. Regions in which all effects are indicated with a dash, the final model solely included framewise displacement as an effect. Significance level: *q < 0.05.

LH, Left Hemisphere; RH, Right Hemisphere; ROIs, Regions of Interest.

**Figure 3:**
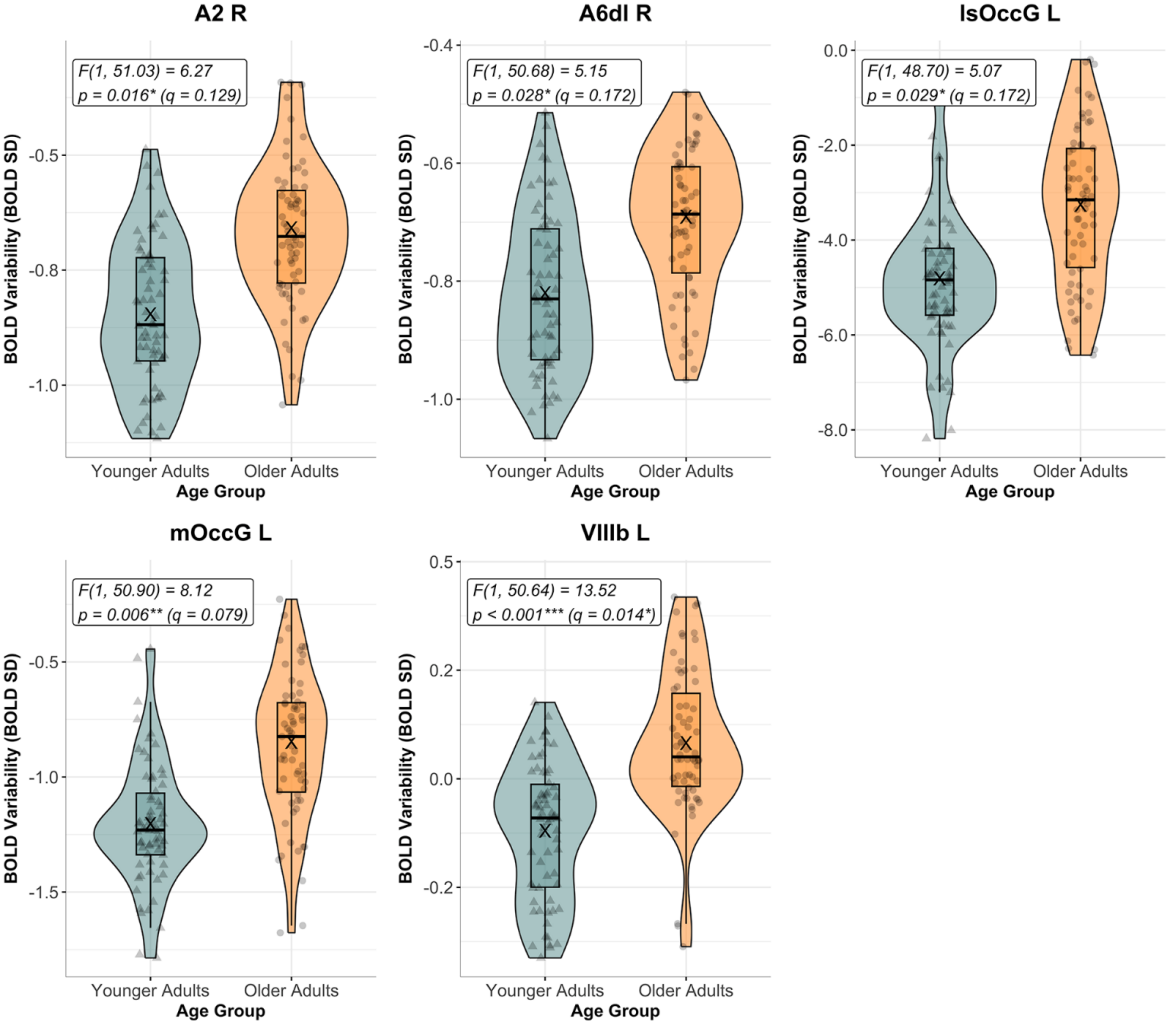
**BOLD variability by age group in significant *ROIs* for main effect of age group before FDR correction.** The figure shows transformed BOLD variability (BOLD SD) across age groups (Younger Adults in teal, and Older Adults in orange) during the Bimanual Tracking Task (BTT) for regions of interest (ROIs) that showed a significant main effect of AGE GROUP prior to FDR correction for multiple comparisons (*p* < 0.05). From these, only the group differences in the cerebellar lobe VIIIb survived correction (with *q* > 0.05). Individual data points reflect BOLD SD values pooled across all task conditions, per subject. F- and p-values for the main effect of AGE GROUP are reported for each ROI. Uncorrected significance levels: * *p* < 0.050, ** *p* < 0.010, *** *p* < 0.001. Corrected significance level: q < 0.05. Abbreviations: A2 = primary somatosensory cortex; A6dl = dorsolateral premotor cortex; FDR = False Discovery Rate; L = left; lsOccG = lateral superior occipital gyrus; mOccG = middle occipital gyrus; R = right; VIIIb = cerebellar lobule VIIIb.

Out of the 25 ROIs analyzed, five showed a significant main effect of AGE GROUP before FDR correction. In all of these regions - namely, right A2, right A6dl, left lsOccG, left mOccG, and left VIIIb – older adults exhibited greater BOLD SD compared to younger adults (see Figure 3; right A2: F[1, 51.03] = 6.27, *p* = 0.016, *β* [Younger] = -0.10; right A6dl: F[1, 50.68] = 5.15, *p* = 0.028, *β* [Younger] = -0.08; left lsOccG: F[1, 48.70] = 5.07, *p* = 0.029, *β* [Younger] = -0.95; left mOccG: F[1, 50.90] = 8.12, *p* = 0.006, *β* [Younger] = -0.24); left VIIIb: F[1, 50.64] = 13.52, *p* < 0.001, *β* [Younger] = -0.18). After correcting for multiple comparisons (with Benjamini-Hochberg FDR method), only the effect in the left VIIIb (*q* = 0.014) remained significant at *q* < 0.05.

Regarding TASK CONDITION, uncorrected effects were found in the left A6dl, left A7r, and left lsOccG, with BOLD SD generally scaling up with task difficulty (i.e., Line 1:1 < Line 3:1 < Angle 3:1) in both age groups (left A6dl: F[2, 84.08] = 3.41, *p* = 0.038, *β* [Line31] = 0.02 and *β* [Angle31] = 0.05; left A7r: F[2, 89.36] = 4.43, *p* = 0.015, *β* [Line31] < 0.01 and *β* [Angle31] = 0.49; left lsOccG: F[2, 48.70] = 5.07, *p* = 0.011, *β* [Line31] = 0.30 and *β* [Angle31] = 0.02). However, none of these effects survived FDR correction (with significance set at *q* < 0.05).

An uncorrected interaction between AGE GROUP and TASK CONDITION was found in six ROIs: right A7m, bilateral V5/MT+, bilateral Crus I, and right VI (right A7m: F[2, 91.08] = 3.14, *p* = 0.047, *β* [Younger; Line31] = -0.05 and *β* [Younger; Angle31] = 0.02); left V5/MT+: F[2, 87.13] = 4.17, *p* = 0.019, *β* [Younger; Line31] = -0.02 and *β* [Younger; Angle31] = 0.08; right V5/MT+: F[2, 90.26] = 3.53, *p* = 0.033, *β* [Younger; Line31] = -0.08 and *β* [Younger; Angle31] = 0.02; left Crus I: F[2, 89.70] = 3.98, *p* = 0.022, *β* [Younger; Line31] = -0.03 and *β* [Younger; Angle31] = 0.03; right Crus I: F[2, 88.92] = 4.73, *p* = 0.011, *β* [Younger; Line31] = -0.04 and *β* [Younger; Angle31] = 0.08; right VI: F[2, 89.95] = 3.99, *p* = 0.022, *β* [Younger; Line31] = -0.03 and *β* [Younger; Angle31] = 0.05). None of the interaction effects survived FDR correction.

**Figure 4:**
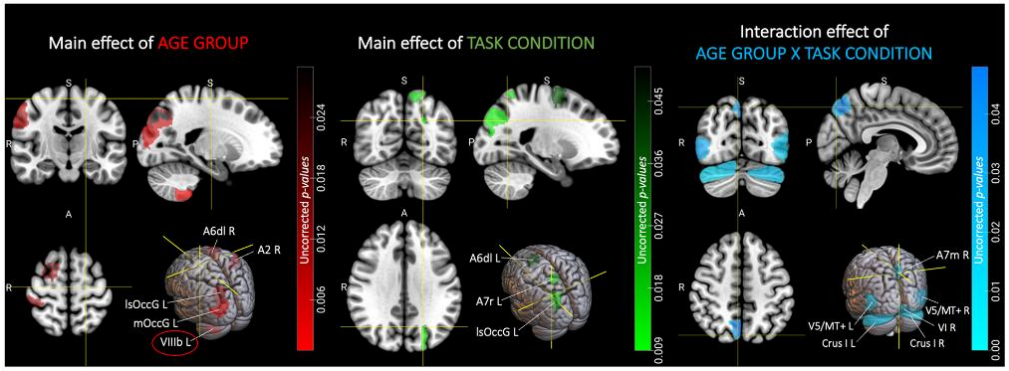
**Regions showing significant effects of age group, task condition and their interaction on BOLD variability prior to FDR correction.** The figure shows, separately for the main effects of AGE GROUP and TASK CONDITION, as well as their interaction effect, the location of the brain regions that yield a significant result prior to FDR correction. Only the effect of AGE GROUP in the left cerebellar lobule VIIIb (circled region) remained significant after correction for multiple comparisons (with *q* < 0.05). Color gradients indicate uncorrected *p*-values for the corresponding effect (red for main effect of AGE GROUP, green for main effect of TASK CONDITION and blue for their interaction) in a given region. Brain slices and volumes depicted in this figure were created with MRIcroGL (Version 1.2.20220720b; https://www.nitrc.org/projects/mricrogl) [37]. Abbreviations: A2 = primary somatosensory cortex; A6dl = dorsolateral premotor cortex; A7m = precuneus; A7r = superior parietal lobe; Crus I = cerebellar lobule Crus I; FDR = False Discovery Rate; L = left; lsOccG = lateral superior occipital gyrus; mOccG = middle occipital gyrus; R = right; V5/MT+ = motion area V5/MT+; VI = cerebellar lobule VI; VIIIb = cerebellar lobule VIIIb.

**Figure 5:**
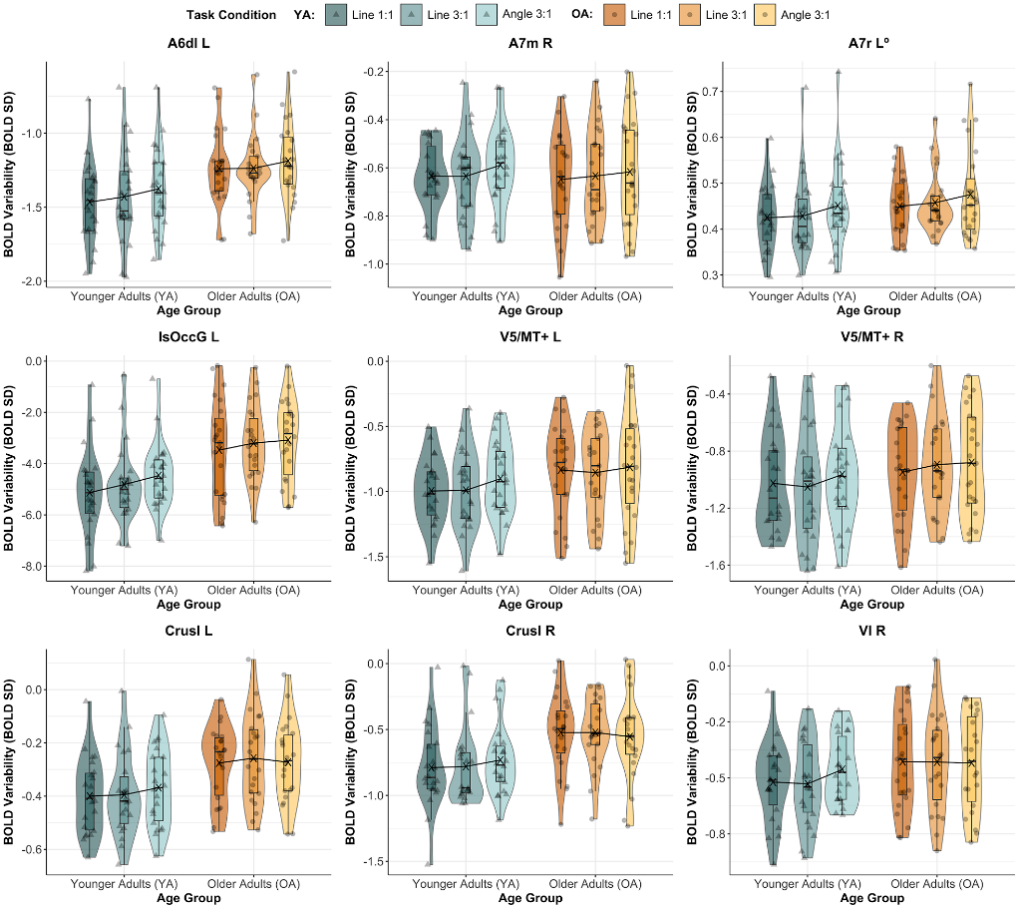
**BOLD variability by age group and task condition in several ROIs.** Figure shows BOLD variability transformed (except for A7r L, as indicated with “^0^”) across age groups (Younger Adults in varying shades of teal, and Older Adults in different shades of orange) and task conditions (Line 1:1, Line 3:1 and Angle 3:1) for ROIs with a significant main effect of TASK CONDITION (namely, A6dl L, A7r L and lsOccG L) or AGE GROUP x TASK CONDITION interaction (i.e., A7m R, bilateral V5/MT+, bilateral Crus I and VI R), before correcting for multiple comparisons. None of these results remained significant after correction (in all, *q* > 0.05). Mean BOLD SD, per group, for each task condition is indicated by an “X”. Abbreviations: A6dl = dorsolateral premotor cortex; A7m = precuneus; A7r = superior parietal lobe; Crus I = cerebellar lobule Crus I; L = Left; L = left; lsOccG = lateral superior occipital gyrus; OA = Older Adults; R = Right; V5/MT+ = motion area V5/MT+; VI = cerebellar lobule VI; YA = Younger Adults.

A visualization of the regions showing significant uncorrected results for each effect, can be found in Figure 4. For a summary of the corresponding results after FDR correction, see Table 2. In Figure 5, the group-specific patterns of BOLD SD change across task conditions are depicted for ROIs that showed a significant uncorrected effect of TASK CONDITION or AGE GROUP X TASK CONDITION.

Lastly, FRAMEWISE DISPLACMENT was consistently a robust predictor across all models, showing a significant positive association with BOLD SD in all ROIs (after FDR correction all ROIs kept a significance of *q* < 0.001), except in left V5/MT+ (*p* = 0.006), right VI (*p* = 0.03), and left VIIIb (*p* = 0.02).

For detailed results of this analysis by ROI, consult Supplementary Table 1.

#### Age group differences in BOLD variability modulation

We found an uncorrected age group difference in BOLD SD modulation across several ROIs (see values in **Bold** in Table 3). These regions included the right A2, right A39c, left A40rd, left lsOccG, left mOccG, right V5/MT+, bilateral Crus I, left VIIb and left VIIIb (right A2: *t* = 3.15, *p* = 0.003; right A39c: *W* = 333, *p* = 0.034; left A40rd: *W* = 353, *p* = 0.009; left lsOccG: *W* = 342, *p* = 0.020; left mOccG: *W* = 336, *p* = 0.028; right V5/MT+: *W* = 316, *p* = 0.040; left Crus I: *W* = 321, *p* = 0.030; right Crus I: *W* = 336, *p* = 0.011; left VIIb: *W* = 398, *p* = 0.001; left VIIIb: *W* = 406, *p* = 0.001). After correction for multiple comparisons, four regions remained significant at *q* < 0.05: the right A2 and the left A40rd (*q* = 0.025), as well as the cerebellar lobules left VIIb and VIIIb (*q* = 0.013). Across these regions, older adults consistently showed more BOLD SD modulation than younger adults, with effects sizes of large magnitude (i.e., Cohen’s d > 0.8 or rank-biserial correlation > 0.5; specifically, right A2: Cohen’s d = -0.96; left A40rd: rank-biserial correlation = -0.53; left VIIb: rank-biserial correlation = -0.57; left VIIIb: rank-biserial correlation = -0.61). A summary of the FDR-corrected results, as well as the descriptive statistics by age group is provided in Table 3.

**Table 3:** Age-group differences in BOLD SD modulation.

**ROIs**		***W*/*t*∇**	***q***	**Rank-biserial correlation/Cohen's d∇**	**Younger adults**			**Older adults**	
					**Median/Mean∇**	**IQR/SD∇**		**Median/Mean∇**	**IQR/SD∇**
RH	A2	3.15∇	0.025*	-0.96 ∇	0.086∇	0.023∇		0.115∇	0.037∇
LH	A37vl	1.61∇	0.184	-0.49 ∇	0.104∇	0.031∇		0.122∇	0.040∇
RH	A39c	333	**0.097**	-0.38	0.087	0.023		0.109	0.032
LH	A40rd	353	0.025*	-0.53	0.074	0.019		0.098	0.044
RH	A5l	310	0.184	-0.28	0.100	0.031		0.106	0.043
LH	A6cdl	299	0.257	-0.24	0.082	0.026		0.087	0.028
LH	A6dl	336	**0.097**	-0.39	0.075	0.016		0.083	0.025
RH	A6dl	332	**0.097**	-0.37	0.093	0.039		0.100	0.025
LH	A6vl	1.75∇	0.157	-0.53 ∇	0.101∇	0.023∇		0.116∇	0.032∇
RH	A7c	235	0.915	0.03	0.115	0.054		0.107	0.034
RH	A7ip	310	0.184	-0.28	0.096	0.028		0.118	0.057
RH	A7m	208	0.491	0.14	0.117	0.050		0.116	0.047
LH	A7r	243	0.991	-0.00	0.090	0.038		0.091	0.035
LH	iOccG	314	0.097	-0.36	0.122	0.047		0.156	0.057
LH	IsOccG	342	**0.081**	-0.41	0.083	0.023		0.099	0.033
LH	mOccG	336	**0.097**	-0.39	0.087	0.020		0.106	0.037
LH	OPC	288	0.357	-0.19	0.131	0.041		0.149	0.075
LH	V5/MT+	257	0.582	-0.13	0.105	0.040		0.108	0.021
RH	V5/MT+	316	**0.097**	-0.37	0.100	0.029		0.121	0.046
LH	Crus I	321	**0.097**	-0.39	0.131	0.051		0.180	0.077
RH	Crus I	336	**0.056**	-0.46	0.124	0.030		0.152	0.052
LH	VI	281	0.301	-0.22	0.144	0.044		0.152	0.059
RH	VI	287	0.357	-0.19	0.135	0.022		0.141	0.040
LH	VIIb	398	0.013*	-0.57	0.120	0.032		0.157	0.104
LH	VIIIb	406	0.013*	-0.61	0.178	0.052		0.229	0.140

Summary of results from analyses examining age-group differences in BOLD SD modulation, for all ROIs, after False Discovery Rate [FDR] correction for multiple comparisons (with significance set at *q* < 0.05). Significance level: * *q* < 0.05. Values in **bold** reflect effects significantly before FDR correction (*p* < 0.05). Descriptive statistics are provided for each age group. For variables violating normality assumptions, medians and interquartile ranges (IQR) are reported and Wilcoxon Rank Sum tests were used. For normally distributed data, means and standard deviations (SD) are reported and independent-samples T-tests were conducted (indicated with “∇”).

Effect sizes are reported as rank-biserial correlations or Cohen’s *d*, depending on whether non-parametric or parametric tests were used for the corresponding age-group comparison.

IQR, Interquartile Range; LH, Left Hemisphere; RH, Right Hemisphere; ROIs, Regions of Interest; SD, Standard Deviation.

Across ROIs, up to one participant per age group was excluded due to BOLD SD modulation values exceeding three standard deviations from their group's mean in that region.

### Impact of age-group differences in BOLD variability and BOLD variability modulation on BTT performance

#### Effect of BOLD variability differences in age groups and task conditions on BTT performance

For each ROI that showed a significant result in any of the previous analyses before FDR correction, we additionally examined whether BOLD variability, task demands, and age group affect behavioral performance.

For that, we employed a LMM per ROI that tested the effects of BOLD SD_REGRESSED_ (i.e., BOLD SD signal regressed for FD), TASK CONDITION, AGE GROUP and the interactions of BOLD SD_REGRESSED_ x TASK CONDITION and BOLD SD_REGRESSED_ x AGE GROUP on BTT SCORE. This analysis revealed an uncorrected positive association between BOLD SD_REGRESSED_ and BTT performance in the right A7m and left lsOccG (right A7m: F[1, 114.54] = 5.20, *p* = 0.025, *β* = 26.57; left lsOccG: F[1, 121.60] = 5.81, *p* = 0.017, *β* = 19.49), although neither effect survived FDR correction (*q* = 0.280 for both regions).

In contrast, AGE GROUP and TASK CONDITION showed consistent main effects in the right A7m, left IsOccG, right Crus I and right VI, with *p*-values < 0.001 that remained significant after correction for multiple comparisons.

With respect to the interaction BOLD SD_REGRESSED_ x TASK CONDITION, only the left A6dl, right A6dl, left mOccG, left V5/MT+, left Crus I, and left VIIIb yielded uncorrected effects (left A6dl: F[2, 92.66] = 3.32, *p* = 0.041, *β* [Line31] = 42.75 and *β* [Angle31] = 72.14; right A6dl: F[2, 92.30] = 5.03, *p* = 0.009, *β* [Line31] = 46.32 and *β* [Angle31] = 65.03; left mOccG: F[2, 96.84] = 3.60, *p* = 0.031, *β* [Line31] = 38.88 and *β* [Angle31] = 13.72; left V5/MT+: F[2, 91.96] = 4.02, *p* = 0.021, *β* [Line31] = 36.57 and *β* [Angle31] = 16.06; left Crus I: F[2, 94.48] = 5.15, *p* = 0.007, *β* [Line31] = 40.76 and *β* [Angle31] = 33.69; left VIIIb: F[2, 91.92] = 3.48, *p* = 0.035, *β* [Line31] = 19.84 and *β* [Angle31] = 16.04). However, none reached the level of significance after FDR correction (*q* = 0.120 in the right A6dl, left V5/MT+ and left Crus I; *q* = 0.125 in the left A6dl, left mOccG and left VIIIb).

Lastly, regarding the BOLD SD_REGRESSED_ x AGE GROUP interaction, a uncorrected effect was found for the right A2, left A40rd and left Crus I (right A2: F[1, 94.60] = 4.00, *p* = 0.048, *β* [younger] = -65.23; left A40rd: F[1, 81.82] = 6.56, *p* = 0.012, *β* [younger] = -75.88; left Crus I: F[1, 100.71] = 4.28, *p* = 0.041, *β* [younger] = -43.34). None of these interaction effects survived correction for multiple comparisons at *q* < 0.05 (right A2r: *q* = 0.328; left A40rd: *q* = 0.196; left Crus I: *q* = 0.123).

Detailed results of this analysis by ROI can be found in Supplementary Table 2.

#### Effect of age-group differences in BOLD variability modulation on BTT performance

To investigate the relationship between BOLD variability modulation, the age group and behavioral performance, we conducted a multiple linear regression for each of the sixteen ROIs (i.e., regions highlighted in **bold** in Table 4). In each model, MEAN BTT SCORE was predicted by BOLD SD MODULATION, AGE GROUP (Younger Adults vs. Older Adults), and their interaction. On average, per ROI, one participant (generally an older adult; corresponding to 2.2% of the total number of participants; ranging from zero to two participants) was excluded from the analysis because BOLD SD modulation was more than three standard deviations from the region’s mean BOLD SD modulation.

**Table 4:** Local maxima and correspondent brain regions in the atlases.

**Brain region**			**Atlas label**	**Local maxima**			
				**Z**	**x**	**y**	**z**
*Brainnetome*							
**LH**	**Superior frontal gyrus**	**A6dl, dorsolateral area 6**	7	5.62	56	65	67
**RH**	**Superior frontal gyrus**	**A6dl, dorsolateral area 6**	8	5.93	35	68	63
LH	Middle frontal gyrus	A6vl, ventrolateral area 6	25	6.08	56	64	61
LH	Precentral gyrus	A6cdl, caudal dorsolateral area 6	55	5.99	62	59	61
LH	Inferior temporal gyrus	A37vl, ventrolateral area 37	97	6.88	70	31	33
**LH**	**Superior parietal lobule**	**A7r, rostral area 7**	125	7.47	51	30	68
				6.5	53	35	68
				6.48	51	35	72
RH	Superior parietal lobule	A7c, caudal area 7	128	6.74	37	26	65
RH	Superior parietal lobule	A5l, lateral area 5	130	5.84	28	45	57
RH	Superior parietal lobule	A7ip, intraparietal area 7	134	5.7	30	38	66
**RH**	**Superior parietal lobule**	**A39c, caudal area 39**	136	5.69	24	31	44
				5.66	21	25	47
				5.65	24	24	50
**LH**	**Inferior parietal lobule**	**A40rd, rostrodorsal area 40**	139	6.08	64	43	58
				5.98	68	45	56
**RH**	**Precuneus**	**A7m, medial area 7**	148	6.91	42	33	65
**RH**	**Postcentral gyrus**	**A2, area 2**	160	6.07	24	49	57
**LH**	**Lateral occipital cortex**	**mOccG, middle occipital gyrus**	199	7.67	59	17	42
				6.97	63	20	38
**LH**	**Lateral occipital cortex**	**V5/MT+, area V5/MT+**	201	7.5	66	27	37
**RH**	**Lateral occipital cortex**	**V5/MT+, area V5/MT+**	202	6.69	21	32	37
				5.77	21	26	39
LH	Lateral occipital cortex	OPC, occipital polar cortex	203	6.45	53	13	40
LH	Lateral occipital cortex	iOccG, inferior occipital gyrus	205	5.7	62	22	30
**LH**	**Lateral occipital cortex**	**lsOccG, lateral superior occipital gyrus**	209	6.92	55	21	57
*Cerebellum SUIT*							
LH	Cerebellum	VI	5	5.86	61	38	18
**RH**	**Cerebellum**	**VI**	7	5.73	32	31	23
**LH**	**Cerebellum**	**Crus I**	8	5.96	65	37	19
**RH**	**Cerebellum**	**Crus I**	10	6.17	22	32	22
				5.67	26	37	20
**LH**	**Cerebellum**	**VIIb**	14	6.4	47	25	16
**LH**	**Cerebellum**	**VIIIb**	20	6.16	51	32	10

Coordinates and Z-values of local maxima spaced at least 8 mm apart, derived from clusters containing 10 or more voxels identified in the functional activation contrast “Angle 3:1 > Line 3:1”, since the contrast “Line 3:1 > Line 1:1” did not show significant clusters of brain activation. Activation peaks were subsequently labeled using the Brainnetome Atlas (for cortical regions) and the Cerebellum SUIT Atlas (for cerebellar regions), resulting in the 25 brain regions indicated (see Figure 9). Only the regions highlighted in **bold** integrated all analyses in the current study (see section 4.8.2.3).

LH, Left hemisphere; RH, Right hemisphere.

After FDR correction, significant main effects of AGE GROUP were consistently observed across all analyzed ROIs (*q* < 0.001), indicating that age remained a strong predictor of BTT performance regardless of BOLD SD modulation.

An uncorrected main effect of BOLD SD MODULATION was found in the left lsOccG, mOccG and Crus I (left lsOccG: *p* = 0.028, *β* = 41.87; left mOccG: *p* = 0.011, *β* = 25.66; left Crus I: *p* = 0.003, *β* = - 38.18). However, only in the left mOccG and left Crus I these effects remained significant after correction for multiple comparisons (*q* = 0.042 and *q* = 0.014, respectively).

**Figure 6:**
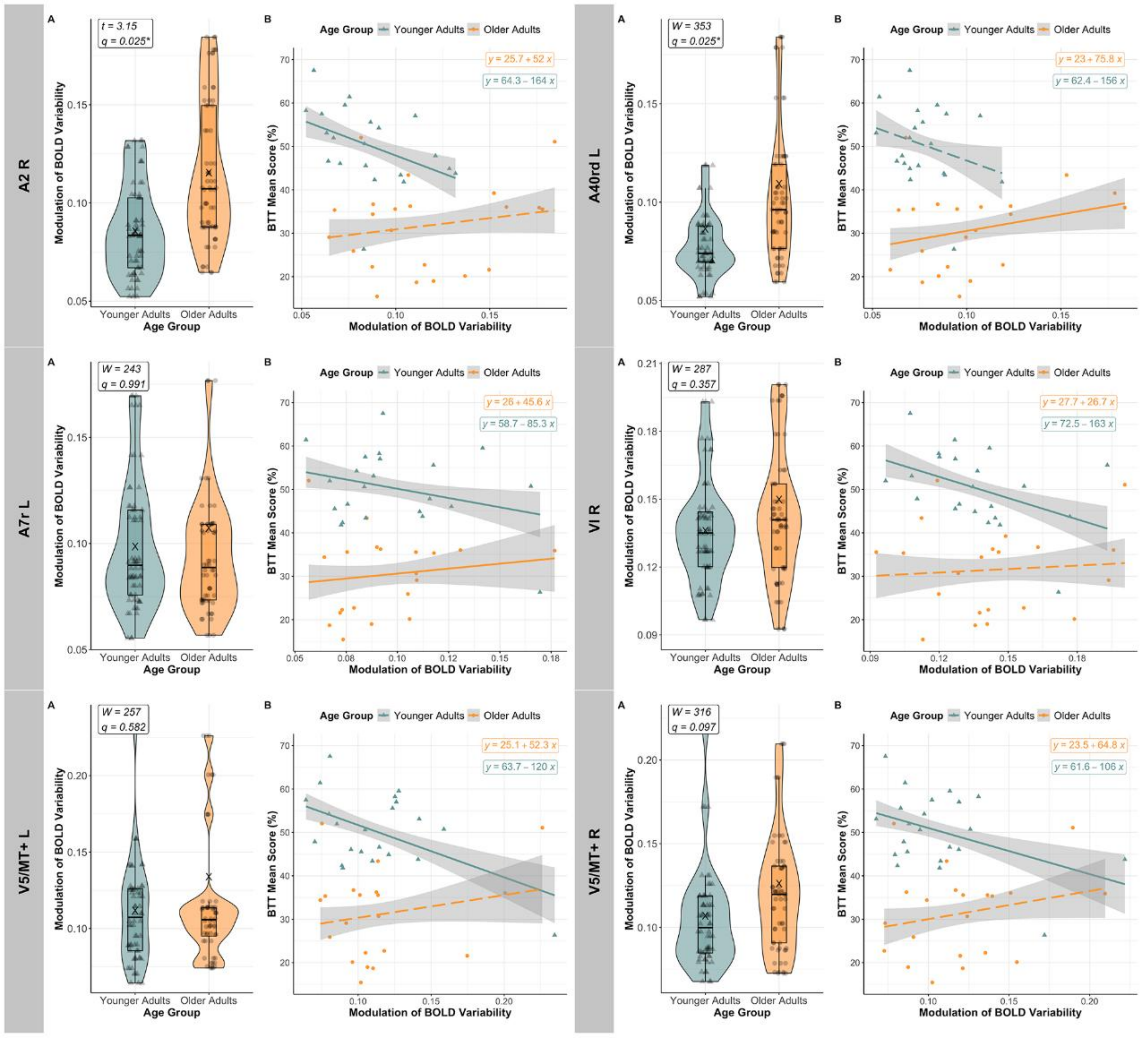
**Age-related modulation of BOLD variability and its relationship with BTT performance.** For each region of interest (ROI) where BOLD SD MODULATION significantly predicted BTT MEAN SCORE after FDR correction in at least one of the age groups, the figure shows: [A] BOLD variability (BOLD SD) modulation across age groups (Younger Adults in teal [triangles], and Older Adults in orange [circles]). In the right primary somatosensory cortex (A2), the left inferior parietal lobe (A40rd), as well as the left cerebellar lobules VIIb and VIIIb (not displayed in the figure), older adults exhibited, on average (“X”), more BOLD SD modulation than younger adults, with *q* < 0.05 (FDR-corrected). Significance level: * *q* < 0.050; [B] The relationship between BOLD SD modulation and mean BTT performance, shown per age-group (Younger Adults again in teal [triangles], and Older Adults in orange [circles]). After FDR correction, younger adults consistently exhibited a significant negative association between BOLD SD modulation and performance across all ROIs analyzed, except for the left inferior parietal lobe (A40rd), while older adults showed a positive relationship in the left inferior and parietal lobes (left A40rd and A7r, respectively). Dashed regression lines identify effects that did not survive FDR correction. Abbreviations: A2 = primary somatosensory cortex; A40rd = inferior parietal lobe; A7r = superior parietal lobe; FDR = False Discovery Rate; L = Left; R = Right; V5/MT+ = motion area V5/MT+; VI = cerebellar lobule VI.

Additionally, FDR-corrected significant AGE GROUP × BOLD SD MODULATION interactions emerged in the right A2, left A40rd, left A7m, left A7r, left V5/MT+, right V5/MT+, and right VI (right A2: *q* = 0.001, *β* [younger] = - 215.52; left A40rd: *q* = 0.002, *β* [younger] = - 123.33; left A7m: *q* = 0.039, *β* [younger] = - 81.95; left A7r: *q* < 0.001, *β* [younger] = - 172.45; left V5/MT+: *q* < 0.001, *β* [younger] = -156.75; right V5/MT+: *q* < 0.001, *β* [younger] = - 168.31; right VI: *q* = 0.003, *β* [younger] = - 191.81). These results suggested that the relationship between BOLD variability modulation and task performance differed by age in these regions (check Figure 6). A summary of the FDR-corrected results for all regions analyzed can be found in Table 5. Detailed results of this analysis for each ROI are presented in Supplementary Table 3.

**Table 5:** Effect of age group, BOLD SD modulation and their interaction on mean BTT score in all ROIs analyzed.

**ROIs**		**Age group**			**BOLD SD modulation**			**Age group X BOLD SD modulation**	
		***F***	***q***		***F***	***q***		***F***	***q***
RH	A2	44.36	< 0.001^***^		2.83	0.182		13.47	0.001**
RH	A39c	131.19	< 0.001^***^		-	-		-	-
LH	A40rd	71.57	< 0.001^***^		18.85	< 0.001^***^		12.60	0.002**
LH	A6dl	131.19	< 0.001^***^		-	-		-	-
RH	A6dl	128.53	< 0.001^***^		-	-		-	-
RH	A7m	39.73	< 0.001^***^		2.49	0.188		5.86	0.039*
LH	A7r	66.43	< 0.001^***^		16.42	< 0.001^***^		17.22	<0.001***
LH	IsOccG	138.17	< 0.001^***^		4.94	**0.064**		-	-
LH	mOccG	143.00	< 0.001^***^		6.74	0.042^*^		-	-
LH	V5/MT+	81.15	< 0.001^***^		6.04	0.042^*^		23.02	<0.001***
RH	V5/MT+	60.89	< 0.001^***^		6.50	0.042^*^		17.58	<0.001***
LH	Crus I	110.74	< 0.001^***^		9.35	0.014^*^		-	-
RH	Crus I	131.19	< 0.001^***^		-	-		-	-
RH	VI	33.23	< 0.001^***^		1.17	0.410		12.17	0.003**
LH	VIIb	123.87	< 0.001^***^		2.90	0.182		-	-
LH	VIIIb	132.90	< 0.001^***^		-	-		-	-

Summary of results from the regression analyses conducted per ROI to investigate whether BOLD SD modulation predicted mean BTT performance (Mean BTT Score) and whether this relationship differed by age group. Values in **bold** reflect significant effects (*p* < 0.05) before FDR correction for multiple comparisons. A dash (“-”) indicates that the corresponding term was excluded from the final model following stepwise simplification. Significance levels: *q < 0.05. **q < 0.01. ***q < 0.001.

LH, Left Hemisphere; RH, Right hemisphere; ROIs, Regions of Interest.

For ROIs where residuals violated normality or homoscedasticity assumptions, we employed residuals bootstrapping (20,000 samples) to obtain robust estimates and 95% confidence intervals. Predictors whose bootstrapped confidence intervals excluded zero were considered statistically significant. Importantly, after applying this residuals’ bootstrapping procedure, the estimated regression coefficients and confidence intervals remained largely consistent with those from the original models (i.e., 95 % CIs [displayed in Table 6] overlapped with the original estimates). This robustness supported the reliability of the observed effects and reinforced the conclusions drawn from the standard analysis.

**Table 6:** Confidence intervals from percentile residual bootstrapping analysis.

**ROIs**		**Age group**		**BOLD SD modulation**		**Age group x BOLD SD modulation**
		**95% bootstrap CIs**		**95% bootstrap CIs**		**95% bootstrap CIs**
RH	A2	[27.33, 49.81]		[-8.21, 111.28]		[-328.50, -100.30]
LH	A40rd	[24.33, 38.98]		[58.60, 154.30]		[-190.90, -55.00]
LH	A6dl	[15.03, 37.93]		[-55.32, 78.22]		[-224.40, 33.70]
RH	A6dl	[15.32, 40.07]		[-80.60, 96.43]		[-215.80, 27.10]
RH	A7m	[20.15, 38.13]		[-8.90, 91.48]		[-146.80, -15.50]
LH	A7r	[27.68, 44.89]		[46.70, 129.30]		[-250.80, -91.40]
LH	IsOccG	[14.74, 33.49]		[10.93, 92.55]		[-135.99, 46.08]
LH	V5/MT+	[28.99, 44.77]		[8.31, 65.49]		[-218.70, -93.50]
RH	Crus I	[9.59, 27.25]		[-41.51, 20.33]		[-55.36, 52.94]
RH	VI	[29.86, 60.37]		[-22.27, 80.11]		[-298.89, -85.69]
LH	VIIIb	[11.99, 37.37]		[-26.20, 16.57]		[-103.25, 28.67]

95 % Confidence Intervals (CIs) based on percentiles for AGE GROUP, BOLD SD MODULATION and their interaction resulting from a bootstrapping analysis of the residuals conducted for each region of interest (ROI) whose residuals violated normality and/or homoscedasticity assumptions.

CIs, Confidence Intervals; LH, Left Hemisphere; RH, Right Hemisphere; ROIs, Regions of Interest.

Lastly, a post hoc simple regression analysis was conducted per age group to determine the relationship between MEAN BTT SCORE and BOLD SD MODULATION in regions showing an FDR-corrected significant AGE GROUP X BOLD SD MODULATION interaction. These analyses revealed that younger adults exhibit a significant negative association between BTT performance and BOLD SD modulation in five out of the seven regions analyzed (namely, right A2, left A7r, bilateral V5/MT+ and right cerebellar lobule VI) after FDR correction (all with q < 0.05). Conversely, in older adults, only in the left A40rd and the left A7r, higher BOLD SD modulation predicted higher BTT mean score before and after FDR correction for multiple comparisons (*q* < 0.001 and *q* = 0.001, respectively). Consult Figure 6 for a visualization of the relationship between BOLD variability modulation and BTT performance per age group, in all the ROIs analyzed. Coefficient estimates and confidence intervals from the bootstrapping analyses validated these same conclusions. Specifically, for younger adults, the 95% bootstrapped confidence intervals were the following: right A2 [- 276.30, - 57.50], left A40rd [- 126.90, 29.33], right A7m [- 107.98, 19.42], left A7r [- 190.20, 31.40], left V5/MT+ [- 180.70, - 18.50], right V5/MT+ [- 203.38, - 23.66] and right VI [- 328.70, - 36.50]. In contrast, for older adults, the confidence intervals were: right A2 [- 45.42, 142.03], left A40rd [32.8, 165.4], right A7m [- 30.90, 126.45], left A7r [19.30, 144.40], left V5/MT+ [- 4.84, 79.76], right V5/MT+ [- 14.01, 134.65], and right VI [- 53.28, 107.19].

**Figure 7:**
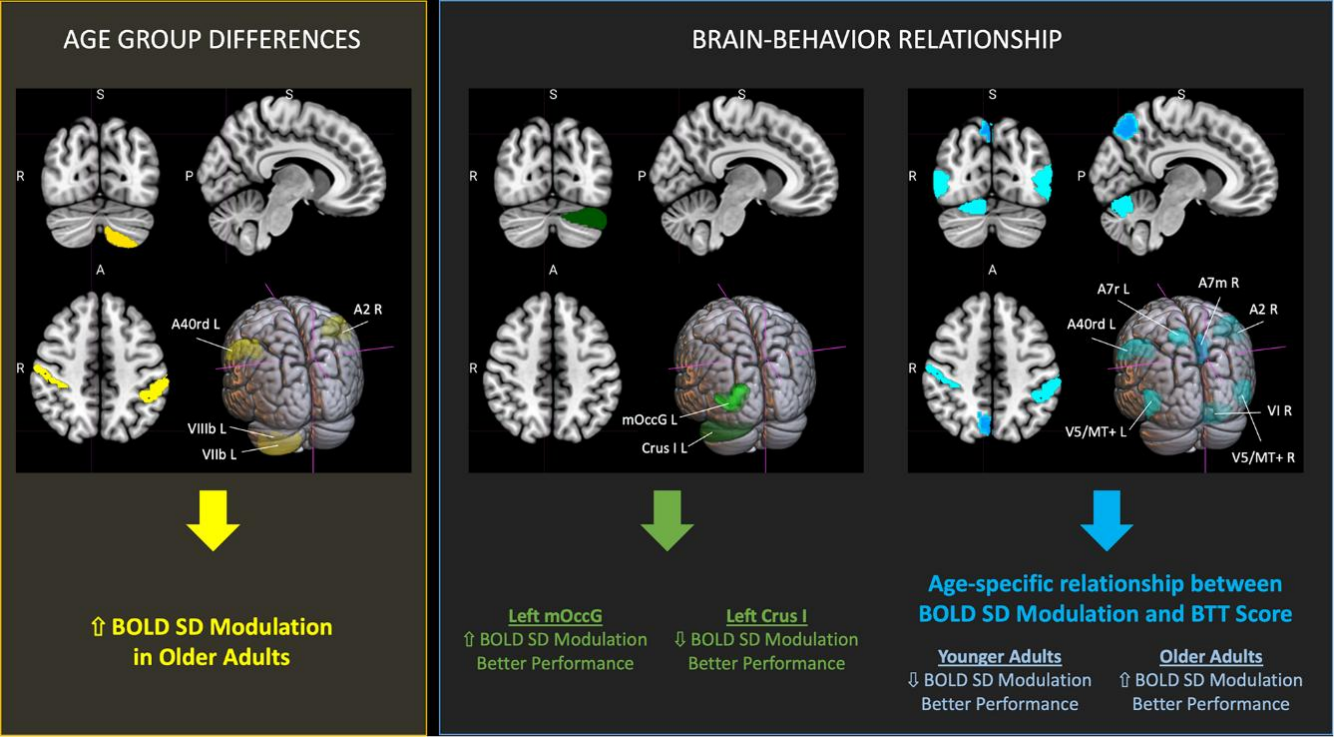
**Significant findings on BOLD SD modulation and its relationship with bimanual task performance in aging.** Summary of brain regions showing significant findings (FDR-corrected, with *q* < 0.05) relative to BOLD SD Modulation and its association with performance on the Bimanual Tracking Task (BTT). Cortical regions were defined based on the Brainnetome Atlas [38], and cerebellar regions using the Cerebellum SUIT Atlas [39]. Brain slices and volumes depicted in this figure in the MNI152 Template were created with MRIcroGL (Version 1.2.20220720b; https://www.nitrc.org/projects/mricrogl) [37]. Abbreviations: A2 = primary somatosensory cortex; A40rd = inferior parietal lobe; A7m = precuneus; A7r = superior parietal lobe; Crus I = cerebellar lobule Crus I; FDR = False Discovery Rate; L = left; mOccG = middle occipital gyrus; R = right; V5/MT+ = motion area V5/MT+; VI = cerebellar lobule VI; VIIb = cerebellar lobule VIIb; VIIIb = cerebellar lobule VIIIb.

An overview of the main findings from the analyses focusing on BOLD SD modulation was depicted in Figure 7.

## DISCUSSION

In the current study, we investigated how BOLD variability - specifically, BOLD SD - and its modulation across different levels of task complexity during a bimanual coordination task are affected by advancing age and relate to task performance.

Contrary to our initial hypotheses, compared with younger adults, older adults exhibited higher BOLD variability in the left VIIIb cerebellar lobule, independent of task complexity, as well as greater BOLD SD modulation in several sensorimotor and cerebellar regions. However, the observed age-related differences in BOLD variability did not predict BTT performance. In contrast, in both age groups, BOLD SD modulation in sensorimotor and visuospatial regions was predictive of BTT score.

Notably, in other regions, the relationship between BOLD SD modulation and BTT performance depended on age group. In particular, in younger adults, reduced modulation consistently predicted better performance across several regions, whereas in older adults, higher modulation in the left inferior and superior parietal lobes - regardless of the direction of modulation (i.e., up- or down-modulation) - was associated with better behavioral outcomes. Together, these findings suggest that advancing age influences the regulation of intrinsic neural dynamics in response to task demands, with implications for bimanual performance.

### Behavioral results: Lower overall BTT performance in older adults, but similar effect of task condition across age groups

Our behavioral analysis revealed robust age-related differences in bimanual performance. Across all conditions of the BTT, younger adults significantly outperformed older adults. Task difficulty also influenced performance: participants performed best on the two line-tracking conditions (Line 1:1 and Line 3:1), and significantly worse on the angle condition (Angle 3:1), indicating that increased complexity impaired task execution. Post hoc comparisons confirmed that performance in the Angle 3:1 condition was significantly lower than in both line-tracking conditions, while no difference emerged between the two “Line” conditions. Importantly, the lack of an interaction between age group and task condition suggests that both younger and older adults were similarly affected by increasing task demands, even though their overall performance levels differed, which contrasts with previous findings indicating that older adults gradually lose their capacity to adapt to varying task requirements [6, 40–42]. This discrepancy between our results and existing literature may stem from differences in the type or amount of feedback provided [43, 44], or even the amount of exposure to the task conditions during the familiarization session. For instance, in all four aforementioned studies, BTT practice during the familiarization session lasted for a fixed duration of six minutes, which may have favored faster-learning in young adults who improved more during the practice, particularly under more challenging task conditions. In contrast, in our study, training was terminated once participants reached a performance threshold of 10%, thereby minimizing initial inter-individual discrepancies in task performance during the MR session. Nevertheless, these findings support the assumption that the Angle 3:1 condition imposed greater cognitive-motor coordination challenges across the lifespan. They also point to a graded behavioral pattern aligned with task complexity, as performance gradually declined from Line 1:1 to Line 3:1 and then more sharply in the Angle 3:1 condition. Although the difference between the two “Line” conditions did not reach statistical significance, the observed trend is consistent with a complexity-driven performance gradient that may help contextualize neural variability modulation.

### Higher BOLD variability in older adults, but no association with BTT performance

While previous cognitive studies reported reduced task-related BOLD variability with aging [14, 15, 34], we found higher task-related BOLD SD in older relative to younger adults in the left VIIIb. This subdivision of the cerebellar lobule VIII is located inferiorly on the posterior cerebellum, and is known to be connected with the somatosensory cerebral network [45], assisting the primary motor cortex (M1) [46] in the execution and coordination of fine limb movements [47], containing, in particular, the somatomotor representations of individual left fingers [48]. This divergence between our results showing higher BOLD SD in older relative to younger adults, and findings from existing literature reporting the opposite pattern, reinforce the notion that variability-age relationships might be task-dependent [27, 31, 32, 35].

Nevertheless, given prior work from Scarapicchia et al. [31] reporting a positive relationship between white matter hyperintensities (WMH) and BOLD variability, particularly in the cerebellum, we speculate that the increase in BOLD variability we observed with aging in the left cerebellar lobule VIIIb might have a vascular or structural origin. It might as well be driven by an age-related disruption of the dopaminergic neuromodulation systems, as lower dopamine has been previously linked to greater BOLD variability, especially in subcortical structures such as the cerebellum [28, 29]. However, the current study did not examine the role of WHM nor of dopaminergic differences in BOLD variability. Hence, future research should investigate these factors as potential mechanisms underlying BOLD variability shifts with aging.

Regarding the potential behavioral relevance of these age-related differences in BOLD variability, we did not find robust evidence supporting a brain-behavior relationship in bimanual performance. This stands in contrast to prior findings in cognitive domains linking BOLD variability to performance [14, 27].

### Age-group differences in the magnitude of BOLD variability modulation predict BTT performance

Our data revealed greater BOLD variability modulation across BTT conditions in older compared to younger adults within the right primary somatosensory cortex (A2), left inferior parietal lobule (A40rd) and left cerebellar lobules VIIb and VIIIb. This pattern diverges from prior findings in cognitive domains, where aging has typically been associated with reduced intra-individual brain variability [25, 33, 34], possibly reflecting domain-specific differences in how neural variability is modulated across the lifespan. The right A2 and the left A40rd support the integration of sensory input necessary to motor planning and execution [49, 50], whereas VIIb and VIIIb are implicated in the active maintenance of attentional processes and working memory [45, 47, 51, 52]. Notably, however, these were not the regions where BOLD variability modulation directly predicted bimanual task performance in the present study.

In addition to age-related differences in BOLD variability modulation, we observed region-specific relationships between BOLD variability modulation and task performance. In the left middle occipital gyrus (mOccG), greater modulation of BOLD variability was associated with better behavioral outcomes, suggesting a beneficial role of dynamic neural adaptation in visual processing [53], possibly reflecting enhanced discrimination of visual features across task condition.

In contrast, in left Crus I - a region previously implicated in higher-order cognitive processes crucial for managing the complex demands of bimanual coordination, particularly under cognitive load [4, 42, 51], such as cognitive control and working memory [34, 36, 45, 51, 52, 54–56] - higher BOLD variability modulation predicted poorer performance. One possible interpretation is that individuals who manage a broad range of motor demands more efficiently may rely on relatively stable cognitive control processes across task conditions, potentially reflecting a comparable level of automaticity [43], and consequently, reduced condition-specific modulation of variability in this region. Alternatively, increased modulation of BOLD variability in the left Crus I may be indicative of maladaptive recruitment of cognitive control processes during task execution [57]. These interpretations remain speculative and warrant confirmation in future studies specifically designed to test these relationships.

A key finding of our study is that BOLD SD modulation differentially influenced BTT performance across age groups. In younger adults, lower BOLD SD modulation in the right primary somatosensory cortex (A2), left superior parietal lobule (A7r), bilateral motion-sensitive visual areas (V5/MT+), and right cerebellar lobule VI was associated with better task outcomes. In contrast, in older adults, higher BOLD SD modulation in the left inferior and superior parietal lobules (A40rd and A7r) predicted better performance. This indicates that the neural dynamics supporting successful task execution may be age-specific, with younger and older adults recruiting and modulating distinct networks to meet task demands.

Drawing on recent literature [42, 49, 50, 57, 58], we propose that better performance in younger adults is linked to reduced BOLD variability modulation in sensorimotor and visuospatial regions because they likely possess well-established motor representations that require minimal recalibration through sensory feedback and motor planning, possibly due to greater task automatization. Conversely, in older adults, our results suggest better task performance is associated with greater adaptation of the neural dynamics in regions involved in executive control, motor attention and visuospatial integration [44, 59]. This potentially reflects the need for more active motor control in the absence of automatization. In this context, the observed increase in BOLD variability modulation across BTT conditions may represent heightened neural adaptability, enabling the dynamic integration of sensory input and top-down control processes necessary for real-time behavioral adjustments.

Existing cognitive aging models have been developed primarily based on mean BOLD activation measures, making it challenging to directly extrapolate their principles to BOLD variability findings. Nevertheless, we tentatively propose that the aforementioned age-specific shift in the relationship between brain dynamics and behavioral performance aligns with the STAC [10] and CRUNCH models [11], despite most group differences in BOLD SD modulation in the corresponding regions did not survive FDR correction (potentially due to the severity of the correction, see Figure 6). These frameworks posit that aging is accompanied by compensatory recruitment of additional higher-order neural resources to maintain task performance. Accordingly, in older adults, heightened up-regulation of BOLD SD with task complexity, particularly within the left inferior and superior parietal lobules (A40rd and A7r), may serve as a compensatory feedback-based strategy [42], potentially expressing increased reliance on visual processing and imagery [58], as well as strategic executive processes [3, 4, 7, 8, 59], to attain the same adaptive efficiency, as achieved by younger adults, through more automatized sensorimotor routines [42, 58]. Behaviorally, both age groups were similarly affected by the task conditions, strengthening our interpretation that successful adaptation to task demands is maintained through age-dependent neural strategies, characterized by specific profiles of BOLD variability modulation.

### Limitations and future directions

Several limitations warrant consideration. First, the absence of direct measurements of vascular reactivity, WMH burden, or dopaminergic function limits our ability to attribute observed effects to specific physiological mechanisms. Without such data, interpretations regarding neural compensation, structural decline, or neurotransmitter shifts remain speculative.

Second, while our sample size was comparable to similar fMRI studies, the relatively small number of participants may have reduced our power to detect subtle effects, especially after correction for multiple comparisons.

Third, the use of a ROI-based approach, while justified statistically, may have masked finer-grained or network-level effects that could have emerged from a voxel-wise or connectivity-based analysis. Moreover, the choice of these regions based on activation maps may have overlooked regions where variability plays a prominent role despite minimal relevance of the average BOLD signal, given the reported orthogonality of mean and variability measures [16]. Therefore, an alternative ROI selection method, as well as whole-brain or network-level approaches should be considered in future work.

Furthermore, parametric task complexity levels were achieved by simultaneously manipulating two task components: speed of rotation and directional shift, making it difficult to determine how each is independently affected in aging, and their corresponding neural correlates. Similarly, the currently used BTT score does not allow to fully dissociate the behavioral component of speed from accuracy. Alternative BTT scoring methods may prove valuable in future research to better establish links between age-specific performance strategies and the modulation of BOLD signal variability.

Notably, although older adults exhibited greater head motion than younger adults during task performance, which can inflate BOLD SD estimation, we mitigated this unwanted effect by accounting for any post-preprocessing residual FD-related variability in our analyses, as advised by [60]. No additional correction was applied in the analyses concerning BOLD SD modulation, as these metric measures intra-individual variability across task conditions and we did not find significant differences in FD across task conditions that justified such correction. Moreover, given that FD variability might also index other confounds such as physiological noise (e.g., respiration, heart rate) [60, 61], by including it in our statistical models, we likely also minimized this source of variability. Nonetheless, although we implemented a variety of steps to control for these potential confounders, we cannot discard with full certainty the possibility of remaining residual effects on BOLD variability estimation.

Future research should aim to replicate and extend these findings by incorporating multimodal imaging techniques - such as diffusion tensor imaging, arterial spin labeling, positron emission tomography and magnetic resonance spectroscopy to better understand the vascular, structural, and neurochemical correlates of BOLD variability. Longitudinal designs will be particularly valuable in determining whether patterns of variability modulation represent dynamic states shaped by aging, experience, or intervention. Additionally, examining both cognitive and motor domains within the same participants may help clarify whether the neural dynamics of variability modulation are domain-specific or reflect broader, cross-functional signatures of aging while simultaneously shedding light onto the neural correlates of the extensively reported age-related penetration of cognition into action.

## CONCLUSION

In sum, this study highlights the potential role of BOLD variability modulation in shaping bimanual performance during aging. While younger adults appear to benefit from neural stability, older adults may rely on dynamic modulation of variability, particularly in visuospatial and cerebellar networks, to compensate for declining efficiency. These findings complement existing neurocognitive aging theories by identifying BOLD variability modulation as a potential biomarker of neural adaptability and resilience, with relevance for maintaining sensorimotor function in later life.

## MATERIALS AND METHODS

### Participants

The study included 22 younger healthy adults (YA; 10 males, mean ± SD [range]: age 31.27 ± 5.42 [25–40] years) and 23 older healthy adults (OA; 12 males, mean ± SD [range]: age 69.52 ± 4.15 [65–79] years), all recruited from Flanders, Belgium. Participants were recruited via social media, as well as posters and flyers placed in locations relevant to the target population, such as universities, senior homes, sports facilities, and hospitals. All participants were right-handed based on the Edinburgh Handedness Inventory Laterality Quotient (EHI LQ; mean ± SD [range]: 94.87 ± 11.03% [50-100]; cutoff score ≥ 50) [62], were fluent Dutch speakers, and had normal or corrected-to-normal eyesight. Cognitive screening was conducted using the Montreal Cognitive Assessment (MoCA; mean ± SD [range]: 28 ± 1.32 [26-30], cutoff score ≥ 26/30) [63]. Depressive symptoms were assessed using age-specific neuropsychological instruments: the Beck’s Depression Inventory – Second Edition (BDI-II; median ± interquartile range [IQR; range]: 3 ± 4.75 [0-12], cutoff score > 13/20) [64] for younger adults, and the 15-item Geriatric Depression Scale (GDS-15; median ± IQR [range]: 1 ± 1 [0-12], cutoff score > 5/15) [65] for older adults.

Exclusion criteria included self-reported neuropsychiatric disorders (e.g., epilepsy or stroke), history of brain injury or surgery, substance abuse (alcohol or drugs), regular use of psychoactive medication (e.g., antidepressants, sedatives), compromised upper limb mobility, or contraindications for magnetic resonance (MR; e.g., cardiac pacemaker, aneurysm clip, metal prosthesis, claustrophobia). Additionally, individuals with a high level of bimanual proficiency (i.e., engaging in specific bimanual tasks for more than five hours per week, as in [66]), were excluded to ensure a comparable level of bimanual skills across participants at baseline.

Prior to enrolling, participants provided written informed consent. The study protocol was approved by the Ethics Committee Research of UZ/KU Leuven (study number S66028) and complied with the ethical principles regarding research with humans stipulated in the latest amendment of the Declaration of Helsinki [67].

### Experimental protocol

The experimental protocol consisted of two sessions, each of about two hours. The first session was divided into two parts: (1) a comprehensive eligibility screening using standard neuropsychological tests and questionnaires, and (2) a familiarization session with the task used in the current study to assess bimanual coordination, the Bimanual Tracking Task (BTT).

For the screening, participants completed the MoCA [63], EHI [62], BDI-II [64], GDS-15 [65], and a questionnaire assessing MRI exclusion criteria. A summary of the group average scores at each test can be found in Table 7.

**Table 7:** Participants’ demographics.

**Measure**	**Younger Adults**			**Older Adults**			**Group comparison**	
	***Median***	***IQR***		***Median***	***IQR***		**W**	***p*-value**
MoCA (/30)	29	2.00		29	2.00		218.5	0.427
EHI LQ (%)	100	11.88		100	0		283	0.374
BDI-II (/63)	3	4.75						
GDS-15 (/15)				1	1			

Descriptive statistics are provided for each age group based on scores for the tests administered to attest eligibility. Given that data consistently violated the assumption of normality, medians and interquartile ranges (IQR) are reported and Wilcoxon Rank Sum tests were used. No group comparisons were performed for depressive symptoms as each age group completed a different test (BDI-II for younger adults; GDS-15 for older adults). Results indicate that the age groups did not differ in general cognition or handedness. Significance level: *** *p* < 0.001.

BDI-II, Beck Depression Inventory – Second Edition; EHI LQ, Edinburgh Handedness Inventory Lateralization Quotient; GDS-15, Geriatric Depression Scale – 15 Items; IQR, Interquartile Range; MoCA, Montreal Cognitive Assessment.

**Figure 8:**
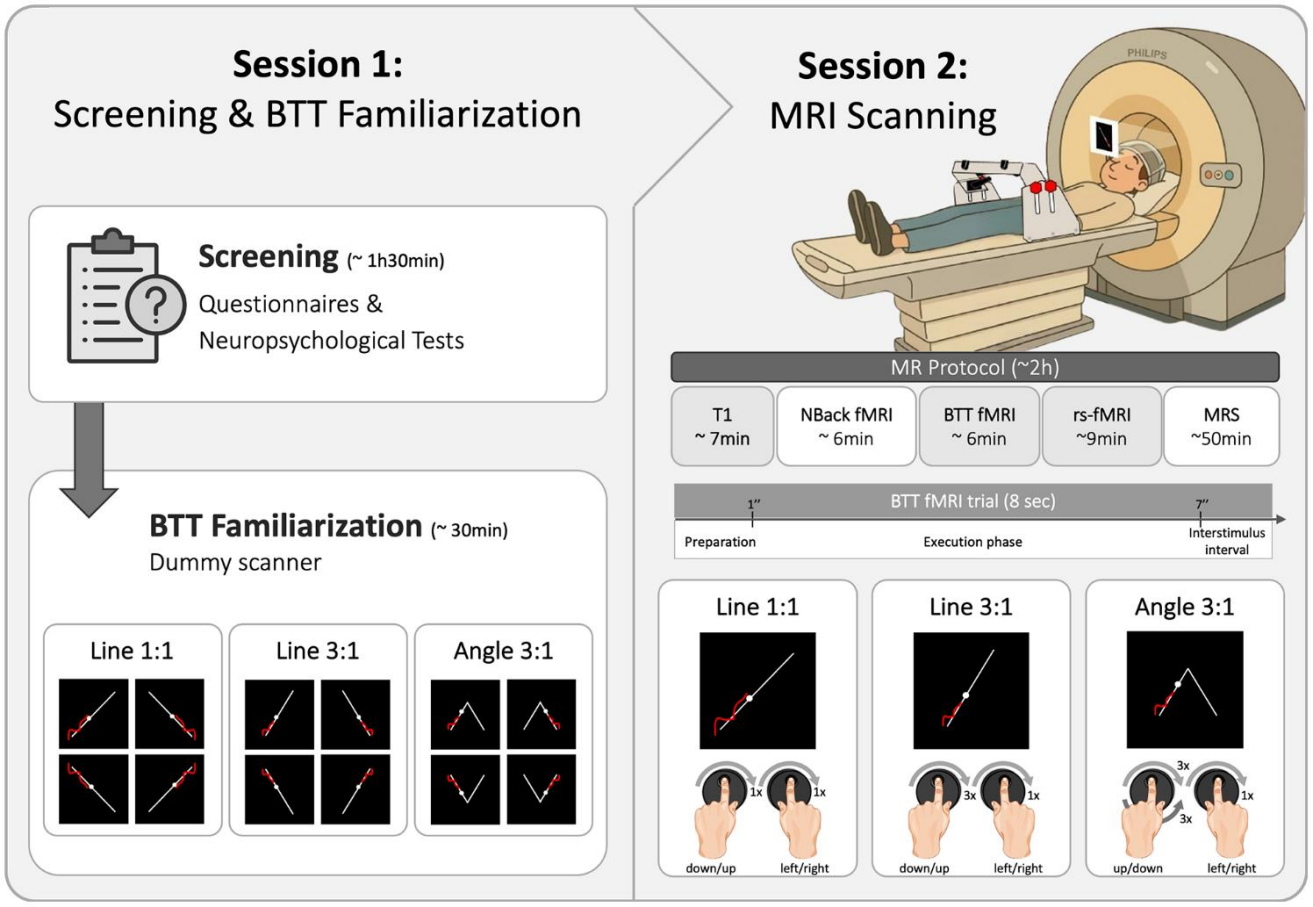
**Experimental protocol.** The figure shows the study protocol consisting of two sessions, each lasting approximately two hours. The first session included a screening and BTT familiarization in a dummy MR scanner. During this session, participants were introduced to the Bimanual Tracking Task (BTT) and performed three task conditions - Line 1:1, Line 3:1, and Angle 3:1 - in all possible directions. The task setup was placed over the participants' hips while they lay supine in the scanner. In the second session, participants underwent an MR protocol comprising several sequences. Each trial of the BTT during fMRI included a 1-second preparation phase, a 6-second execution phase, and a 1-second interstimulus interval. The same three BTT conditions were used during the fMRI scan, but only in a single direction, as shown in the figure. Abbreviations: BTT = Bimanual Tracking Task; fMRI = functional Magnetic Resonance Imaging; MR = Magnetic Resonance; MRS = Magnetic Resonance Spectroscopy; rs-fMRI = resting-state fMRI; T1 = T1-weighted.

During the familiarization session, participants practiced the BTT for eight blocks or until they reached a minimum performance score of 15% in a dummy magnetic resonance (MR) scanner. Each practice block included the same movement patterns as those later performed in the real MR scanner (i.e., Line 1:1, Line 3:1 and Angle 3:1 [left:right hand movements]; see details in section 4.4), but each condition was presented in all four possible directional combinations of dial rotation (Figure 8). This session aimed to ensure participants understood the task instructions in a simulated MRI environment and to minimize the effects of early skill learning.

In the second experimental session, participants completed an extensive imaging protocol consisting of a 7-minute high-resolution T1-weighted structural scan, a 9-minute resting-state functional MRI (fMRI) image, two 6-minute task-related fMRI images (namely, BTT and N-back), and several magnetic resonance spectroscopy (MRS) scans (which took approximately 50 minutes). MR data acquisition took place at the University Hospital of KU Leuven. For the current paper, only data concerning the structural and the functional BTT images were examined.

### Imaging acquisition

All MRI data were collected using a Philips Achieva DStream 3-Tesla scanner equipped with a 32-channel receiver coil. Functional task-related data for the BTT were acquired using a T2*-weighted Echo Planar Imaging (EPI) sequence with the following parameters: repetition time (TR) = 1400 ms, echo time (TE) = 33 ms, flip angle = 70°, voxel size = 2.5 × 2.5 × 2.5 mm³, field of view (FOV) = 240 × 240 × 150 mm³, matrix size = 96 × 95, 60 transverse slices acquired in a sequential ascending order, SENSE/Multiband SENSE factors = 1.5/3, phase-encoding direction = posterior-anterior. A total of 258 dynamic scans were acquired, preceded by 7 dummy scans to allow for magnetic field stabilization. Additionally, high-resolution anatomical images were obtained using a T1-weighted (T1w) Magnetization Prepared Rapid Gradient Echo (MPRAGE) sequence with the following parameters: TR = 5.9 ms, TE = 2.6 ms, flip angle = 8°, voxel size = 0.8 × 0.8 × 0.8 mm³, FOV = 256 × 240 × 166.4 mm³, matrix size = 320 × 300, and 208 transverse slices.

### fMRI task description

A nonferromagnetic version of the Bimanual Tracking Task (BTT) was used to assess visuomotor bimanual coordination during the task-related fMRI scan [68]. This setup, consisting of two handles and two rotational dials (5 cm diameter), was designed to be placed over the participants’ hips, allowing them to perform the task while lying supine in the MR scanner. The task was projected onto a screen positioned at the back of the scanner, and made visible to participants via a mirror (~14 × 9 cm).

BTT performance required the coordinated rotation of two dials using both index fingers, each positioned in a circular groove on the respective dial and controlling either horizontal (x-axis) or vertical movement (y-axis). The objective was to track a white target dot as it moved along a predetermined white trajectory at a constant speed. Participants’ real-time movement trajectory, generated by dial rotation, was displayed as a red line, providing concurrent performance feedback.

BTT conditions differed in inter-hand frequency ratios and/or rotational direction (i.e., clockwise or counterclockwise) of each dial, thereby varying in complexity. Frequency ratios refer to the relative speed of each index finger’s movement. In a 1:1 ratio, both fingers moved at the same speed (i.e., iso-frequency movement), whereas in a 3:1 ratio, the left finger moved three times faster than the right (i.e., non-iso-frequency movement). In this study, three task conditions were used: Line 1:1, Line 3:1, and Angle 3:1 (left:right hand movements). In “Line” conditions, both dials maintained the same rotational direction throughout the entire trial duration (clockwise in this experiment). In contrast, “Angle” conditions required a mid-trial change in the rotational direction of the dial controlling vertical movement (in this study, the left dial). These BTT conditions were chosen based on prior work from our research group that examined how different age groups coped with increasing task complexity using the same task [6, 42].

The BTT was performed during approximately six minutes in a block design. Each task condition was presented for five non-consecutive blocks (each 24 seconds), with three trials per block, totaling 45 trials (3 BTT conditions x 5 blocks x 3 trials/block = 45 trials). All trials had a constant duration of eight seconds. Each began with a 1-second preparation phase during which the target trajectory appeared on a black background, followed by a 6-second movement phase where the white dot moved along the target trajectory, and ended with a 1-second inter-trial interval (see Figure 8). The BTT conditions were presented in a fixed order for each participant but varied across participants in a pseudorandomized manner.

### Behavioral outcome measure

Participant performance on the BTT was expressed in a percentage, with 100 % indicating perfect accuracy. The final BTT score (*S*) was derived from the multiplication of two key components (*S* = *P* × *D*): a preliminary score (*P*) reflecting movement accuracy and a distance correction factor (*D*) accounting for deviations from the target trajectory.

The preliminary score (*P*), calculated as described in [41], resulted from dividing the proportion of unique target points successfully covered in the correct order during the participant’s movement trajectory, by the total number of points forming the target trajectory, all multiplied by 100. These unique target points corresponded to those with the minimal Euclidean distance from the actual movement path, considering a sampling rate of 100 Hz (i.e., every 10 ms; see Figure 1D in [41]). Hence, a higher *P* value indicated greater accuracy in following the intended path. However, a limitation of this method was that it did not distinguish between movements that closely follow the target trajectory and those that are parallel to it.

To penalize movement trajectories parallel to the target trajectory over closely following it, a correction factor (*D*) was applied as previously done in studies from our research group [66, 69]. This factor adjusted the preliminary score based on the average distance ($\bar{d}$) between the actual movement path and the target trajectory throughout a trial. The correction was implemented using the formula:


$$D=\left(1-\frac{\bar{d}}{N}\right)$$

Unlike prior studies in which the scaling factor (*N*) was set to 5 [66, 69], in this study it was adjusted to 8 to better account for performance variability across different age groups. This adjusted N was determined by analyzing unpublished independent BTT data from our research group with a similar set of participants, and aimed to mitigate floor and ceiling effects, ensuring a more accurate assessment of skill differences.

### Imaging preprocessing

Following acquisition, all MRI data were converted to BIDS format and preprocessed at the individual level using fMRIPrep 24.0.01 [70, 71], a robust and standardized preprocessing pipeline implemented in Nipype 1.8.6 [72]. The structural T1-weighted (T1w) image was first corrected for intensity non-uniformity (INU) using ANTs 2.5.1 (Advanced Normalization Tools) [73] and subsequently used as the T1w reference. Skull-stripping was performed using ANTs, and the image was segmented into cerebrospinal fluid (CSF), white matter (WM), and gray matter (GM) using FAST from FSL 6.0.5.2 (FMRIB Software Library) [74]. FreeSurfer 7.3.2 [75] was then used to reconstruct brain surfaces. The T1w image was normalized to MNI152NLin2009cAsym space with an isotropic resolution of 2 mm using nonlinear registration with ANTs, utilizing the brain-extracted versions of both the T1w reference and the T1w template.

Functional image preprocessing began with motion correction of BOLD volumes using MCFLIRT [76] from FSL with a reference BOLD image. Susceptibility distortion correction was performed using a fieldmap-less distortion approach (i.e., SyN correction) [77], in which deformation fields were estimated by co-registering the EPI reference to the same-subject T1w reference with its intensity inverted. Functional images were then corrected for slice timing differences with 3DTshift from AFNI 24.1.22 (Analysis of Functional NeuroImages) [78], and the structural and functional images were co-registered using FreeSurfer with boundary-based registration (BBR) [79], which was implemented with 12 degrees of freedom. To address noise and confounding signals, nuisance regressors, including framewise displacement (FD; a measure of frame-to-frame head movement determined from the realignment estimates), DVARS (i.e., spatial standard deviation of the data after temporal differing), and global signals from WM and CSF, were identified using CompCor [80] and Nilearn [81].

After implementing the standard preprocessing steps in fMRIPrep, further temporal denoising was performed using the Denoiser Toolbox (https://github.com/arielletambini/denoiser). This process of temporal detrending involved regressing out the WM and CSF signals, as well as the three translational (trans_x, trans_y, trans_z) and three rotational (rot_x, rot_y, rot_z) head-movement parameters and their temporal derivatives. High-pass filtering was applied to the functional data with a cutoff of 0.006 Hz (~167 seconds) in the BTT fMRI. Finally, spatial smoothing was performed in SPM12 [82] with MATLAB R2022a (version 9.12.0.2009381) [83], using a Gaussian kernel of 6 mm full width at half maximum (FWHM).

### Brain variability measure

After preprocessing, a whole-brain detrended standard deviation map of the BOLD series (BOLD SD) was computed for each BTT condition (i.e., Line 1:1, Line 3:1, and Angle 3:1) and for the total 9-minute rest scan using the Variability Toolbox (VarTbx; https://github.com/LNDG/vartbx) in SPM12 [82]. An additional whole brain BOLD SD map containing the absolute magnitude of change in BOLD SD across BTT conditions (whereafter referred to as BOLD SD modulation) was posteriorly generated by calculating, per voxel, the sum of the absolute differences in BOLD SD between the different task conditions, as described in the following mathematical formula:


$$\begin{array}{cc} Modulation & =\left|BOLDSD_{Line31}-BOLDSD_{Line11}\right|\\ & +\left|BOLDSD_{Angle31}-BOLDSD_{Line11}\right| \end{array}$$

Hence, this composite, between task condition measure was intended to capture the overall amount of change in BOLD SD across all BTT conditions, irrespective of direction (i.e., increase or decreases in BOLD SD). An illustrative example on how to calculate BOLD SD modulation considering the provided formula can be visualized on Supplementary Figure 1.

**Figure 9:**
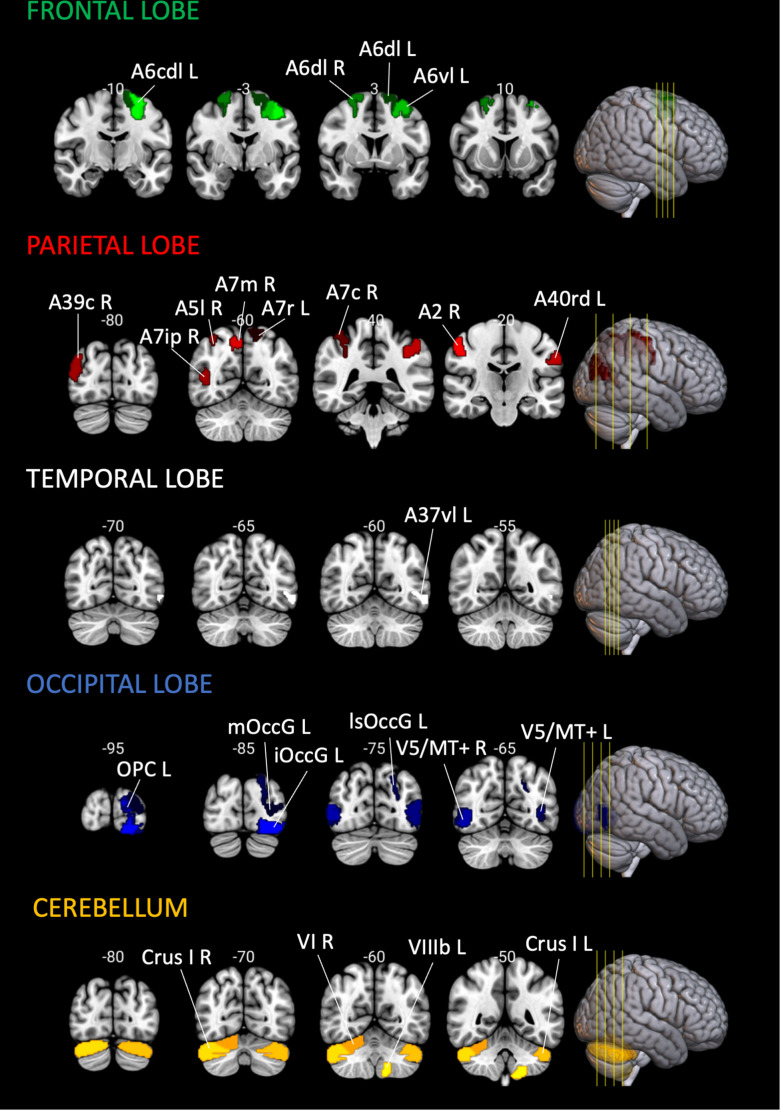
**Location of the brain regions analyzed.** Brain regions analyzed, visualized in the coronal plane, in the MNI152 Template. Cortical regions were labeled using the Brainnetome Atlas [38], and cerebellar regions with Cerebellum SUIT Atlas [39]. Brain slices and volumes depicted in this figure were created with MRIcroGL (Version 1.2.20220720b; https://www.nitrc.org/projects/mricrogl) [37].

In this study, a region-of-interest (ROI)-based approach was chosen over a voxel-wise approach for all statistical analyses given the limited number of subjects per age group (i.e., 22 young adults and 23 older adults). In total, 25 ROIs were analyzed. These regions, listed in Table 4 and displayed in Figure 9, were selected based on a preliminary analysis in FSL, which examined brain activation during BTT performance using independent data from a similar study conducted by our research group. In particular, the included ROIs correspond to local maxima at least 8 mm apart, present in clusters of 10 or more significant voxels after applying a threshold of Z > 5.5 to the contrast Z-maps “Line 3:1 > Line 1:1” and “Angle 3:1 > Line 3:1” (even though no local maxima were found for the contrast map “Line 3:1 > Line 1:1”; thus, consult Table 4 for the coordinates of each local maxima found for the contrast “Angle 3:1 > Line 3:1”). To label the brain regions corresponding to the peak activation coordinates, two widely recognized atlases were used: the Brainnetome Atlas [38] for cortical regions, and the SUIT atlas [39] for cerebellar regions. Subsequently, masks derived from these atlases were created in the same T1w template space and isotropic resolution of 2 mm^3^ as the preprocessed functional images. The resulting masks were applied to the BOLD SD brain maps previously generated per task condition using MATLAB R2022a (version 9.12.0.2009381). For each of these BOLD SD maps, a mean BOLD SD value was extracted per ROI by averaging the BOLD SD across all voxels within the masked regions. Similarly, a mean value of BOLD SD modulation was calculated for each ROI.

### Statistical analyses

All statistical tests were conducted in R (version 4.4.2) [84], with the significance threshold (α) set at 0.05 unless stated otherwise. Prior to analysis, normality was assessed using the Shapiro-Wilk test for the data, and by visually inspecting quantile–quantile (Q-Q) plots for the model residuals. Homoscedasticity was examined using residual-by-predicted plots.

For Linear Mixed Models (LMMs) and Multiple Linear Regressions, model simplification followed a stepwise backward elimination approach, where non-significant terms were sequentially removed, unless they were part of a significant interaction. The significance of model terms was assessed using Type III analysis of variance (ANOVA). When appropriate, post hoc Tukey Honestly Significant Difference tests were performed. Moreover, before running the statistical models, per ROI, datapoints of BOLD SD or BOLD SD modulation more than three standard deviations from the mean were excluded.

To control for Type I error in region-of-interest (ROI) analyses involving multiple comparisons, false discovery rate (FDR) correction was applied using the Benjamini -Hochberg (BH) [85] procedure, with *q* < 0.05 (FDR threshold; *q*-values are FDR-adjusted *p*-values).

### Behavioral data

#### Effect of age group and task condition on BTT performance

BTT behavioral data were recorded and processed online with LabVIEW 8.5 [86]. Subsequent offline data processing was carried out using custom in-house MATLAB scripts (R2023b version 23.2.0.2365128).

To examine differences in BTT score across age groups and task conditions, a 2x3 Mixed Model ANOVA was implemented, with AGE GROUP as a between-subject factor with 2 levels (Younger Adults and Older Adults), TASK CONDITON as within-subjects factor with three levels (Line11, Line31 and Angle31) and BTT SCORE as dependent variable.

### Neuroimaging data

#### Effect of age group and task condition on framewise displacement

Studies such as [60] have shown that BOLD signal remains positively associated with head motion parameters, even after standard denoising steps. Moreover, based on previous research, we expected older adults to exhibit higher average framewise displacement (FD) and anticipated that different task conditions would elicit varying levels of movement-related artifacts. Therefore, to assess the confounding effect of FD on BOLD SD estimations during BTT performance, we tested for FD differences across age groups (younger vs. older adults) and task conditions (Line 1:1, Line 3:1, Angle 3:1) using an Aligned Rank Transform (ART) factorial ANOVA (“ARTool” package) [87], as data did not meet required assumptions to implement an equivalent parametric approach.

### Age-group differences in BOLD variability and BOLD variability modulation during BTT performance

#### Effect of age group and task condition on BOLD variability

To determine how BOLD SD varies by age group and task condition during BTT performance in the selected ROIs, we conducted 25 separate LMMs - one per region - using the “lme4” and “car” R packages [88, 89]. TASK CONDITION (with three levels: Line11, Line31 and Angle31) was included as a within-subjects fixed effect, while AGE GROUP (with two levels: Younger Adults and Older Adults) was treated as a between-subjects fixed effect. In addition, we included in the model the AGE GROUP x TASK CONDITION interaction term, and BOLD SD as the dependent variable. To account for repeated measures, “SUBJECT ID” was modeled as a random intercept, and mean framewise displacement per BTT condition (FRAMEWISE DISPLACEMENT) was included as a covariate of no interest to control for motion-related artifacts in BOLD SD. Thus, the initial model for each ROI was specified in R as: BOLD SD ~ AGE GROUP + TASK CONDITION + AGE GROUP X TASK CONDITION + FRAMEWISE DISPLACEMENT + (1|SUBJECT ID).

When necessary to meet LMM assumptions, BOLD SD was transformed using the Box-Cox transformation (“car” package) [89].

#### Age-group differences in BOLD variability modulation

For each of the 25 ROIs tested in the previous analysis, we additionally examined age-group differences in BOLD SD modulation. Depending on whether the assumptions of normality and homoscedasticity were met, a T-test or a Wilcoxon Rank-Sum Test was employed per brain region. Effect sizes were estimated using Cohen’s *d* or rank-biserial correlations, depending on whether parametric or non-parametric tests were used for the corresponding age-group comparison.

#### Impact of age-group differences in BOLD variability and BOLD variability modulation on BTT performance

To increase statistical power, the following analyses concerning the brain-behavioral relationship between BOLD variability measures (i.e., BOLD SD and BOLD SD modulation) and BTT performance only included brain regions among the initial 25 selected ROIs that presented significant results before multiple comparisons in any of the analyses previously conducted. Therefore, 16 regions (highlighted in **bold,** in Table 4) were analyzed.

#### Effect of BOLD variability differences in age groups and task conditions on BTT performance

A key objective of this study was to examine whether age-group differences in BOLD SD and changes in BOLD SD across different BTT conditions with increasing complexity are associated with BTT performance.

To account for motion-related variability in BOLD SD that might have persisted after denoising during preprocessing, we regressed out mean FD from BOLD SD without centering data around zero (by re-adding the intercept, preserving the original data scale), creating a new variable: BOLD SD_REGRESSED_ (datawizard package) [90]. Posteriorly, per ROI, we conducted the following LMM: BTT SCORE ~ BOLD SD_REGRESSED_ + TASK CONDITION + AGE GROUP + BOLD SD_REGRESSED_ X TASK CONDITION + BOLD SD_REGRESSED_ X AGE GROUP + (1|SUBJECT ID). This means that BOLD SD_REGRESSED_, TASK CONDITON (with three level: Line11, Line31 and Angle31), their interaction (namely, BOLD SD X TASK CONDITION), AGE GROUP (with two levels: Younger Adults and Older Adults), as well as the interaction BOLD SD_REGRESSED_ X AGE GROUP, were included as fixed effects, and SUBJECT ID as random intercept.

#### Effect of age-group differences in BOLD variability modulation on BTT performance

In parallel, for the same 16 ROIs, we also performed a Multiple Linear Regression separately for each ROI to study how the effect of aging on BOLD SD modulation impacts overall BTT performance. Therefore, MEAN BTT SCORE was modeled as a function of BOLD SD MODULATION (check section 4.4 to see how it was calculated), AGE GROUP (with two levels: Younger Adults and Older Adults) and the interaction BOLD SD MODULATION x AGE GROUP. This resulted in the following model: MEAN BTT SCORE ~ BOLD SD MODULATION + AGE GROUP + BOLD SD MODULATION X AGE GROUP.

For ROIs in which residuals were non-normally distributed and/or heteroscedastic, even after data transformation, we employed residuals bootstrapping to obtain more robust coefficients estimates (using “boot” package). This non-parametric method generated 20,000 bootstrap samples by resampling residuals from the original model and adding them back to fitted values. The regression was re-estimated for each sample, and 95% confidence intervals for coefficients were derived from the 2.5^th^ and 97.5^th^ percentiles of the bootstrap distribution. Regressors with confidence intervals excluding zero were considered significantly associated with MEAN BTT SCORE.

Furthermore, upon an interaction effect that remained significant after correction for multiple comparisons, the simple linear regression “MEAN BTT Score ~ BOLD SD MODULATION” was conducted post hoc per age group to examine how the two variables relate in each age group. Once more, when data violated the assumptions of normality and/or homoscedasticity, residuals bootstrapping was performed in the same way as before to determine robust coefficient estimates and compare results from both analyses. *P*-values from the simple regression were corrected with FDR with significance set at *q* < 0.05.

## Supplementary Material

Supplementary Figure 1

Supplementary Table 1

Supplementary Table 2

Supplementary Table 3
