# Supplementary material for: Age-specific relationship between the modulation of brain dynamics in response to task demands and bimanual performance
Source: Aging (Albany NY). 2026 Mar 24;18(1):159–89. doi: 10.18632/aging.206363 (PMC13285952; doi:10.18632/aging.206363)
Supplement: Supplementary Figure 1 [file aging-18-1-206363-s001.pdf]

SUPPLEMENTARY FIGURE

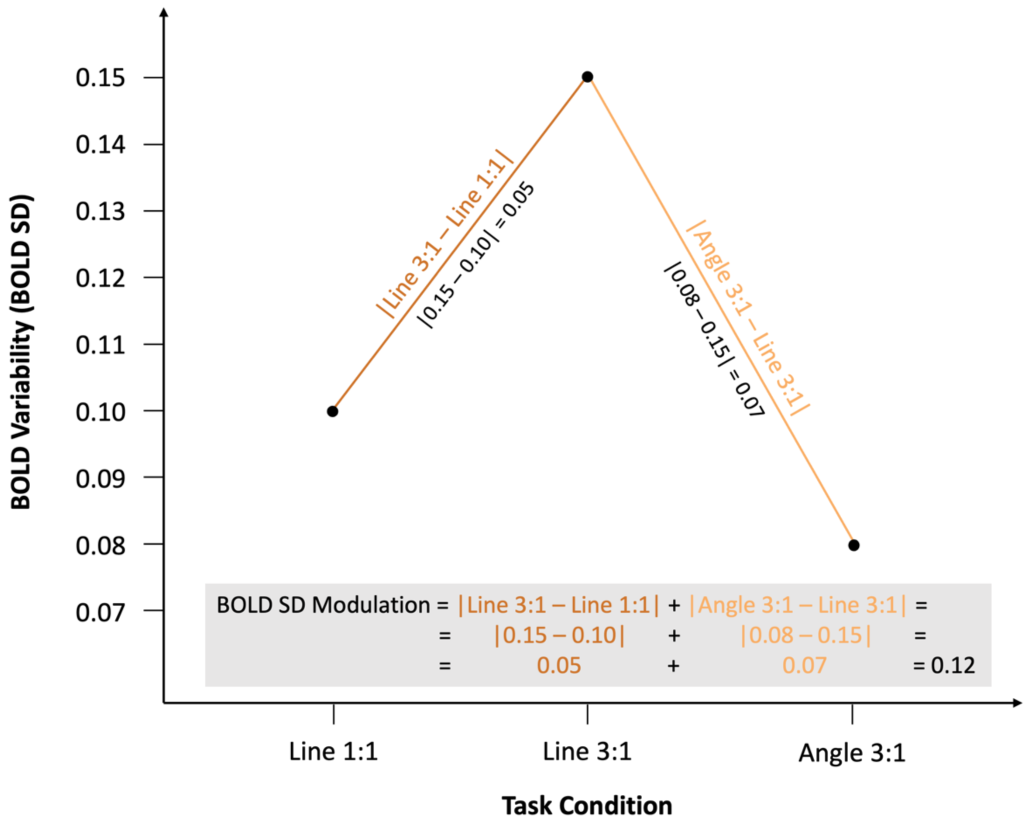

Supplementary Figure 1. Illustrative Example of BOLD SD Modulation Calculation. *Note.* Illustrative example on how to calculate BOLD SD Modulation, a measure of total amount of change across different conditions of the Bimanual Tracking Task (Line 1:1, Line 3:1 and Angle 3:1), based on its mathematical formula.
