# Supplementary material for: Age-specific relationship between the modulation of brain dynamics in response to task demands and bimanual performance
Source: Aging (Albany NY). 2026 Mar 24;18(1):159–89. doi: 10.18632/aging.206363 (PMC13285952; doi:10.18632/aging.206363)
Supplement: Supplementary Table 1 [file aging-18-1-206363-s002.docx]

**Supplementary Table 1. Detailed results from linear mixed model with BOLD variability as dependent variable.**

| Term  [reference level] | | Fixed Effects | | | | ANOVA Type III | |
| --- | --- | --- | --- | --- | --- | --- | --- |
|  |  | $\beta$ | SE | *t* _(df)_ | *p-*value | F-value _(NumDF, DenDF)_ | *p-*value |
| **Right A2**  *BOLDVariability_Trans ~ FramewiseDisplacement + AgeGroup + (1 \| SubjectID)* | | | | | | | |
|  | Intercept | -0.94 | 0.05 | t_(94.56)_=  -17.55 | < 0.001*** |  |  |
|  | Framewise Displacement | 0.84 | 0.14 | t_(115.09)_ = 6.04 | < 0.001*** | F_(1,115)_ = 36.50 | < 0.001*** |
|  | Age Group [Younger Adult] | -0.10 | 0.04 | t_(51.04)_=  -2.50 | 0.016* | F_(1,51.04)_ = 6.27 | 0.016* |
| **Left A37vl**  *BOLDVariability_Trans ~ FramewiseDisplacement + (1 \| SubjectID)* | | | | | | | |
|  | Intercept | -1.14 | 0.09 | t_(105.82)_=  -13.39 | < 0.001*** |  |  |
|  | Framewise Displacement | 0.94 | 0.26 | t_(129.28)_ = 3.65 | < 0.001*** | F_(1,129.28)_ = 13.34 | < 0.001*** |
| **Right A39c**  *BOLDVariability ~ FramewiseDisplacement + (1 \| SubjectID)* | | | | | | | |
|  | Intercept | 0.36 | 0.02 | t_(74.91)_ = 21.21 | < 0.001*** |  |  |
|  | Framewise Displacement | 0.39 | 0.06 | t_(84.23)_ = 6.95 | < 0.001*** | F_(1,84.28)_ = 48.35 | < 0.001*** |
| **Left A40rd**  *BOLDVariability_Trans ~ FramewiseDisplacement + (1 \| SubjectID)* | | | | | | | |
|  | Intercept | -1.54 | 0.09 | t_(103.91)_ =  -17.72 | < 0.001*** |  |  |
|  | Framewise Displacement | 1.27 | 0.26 | t_(127.21)_ = 4.82 | < 0.001*** | F_(1,127.22)_ = 23.20 | < 0.001*** |
| **Right A5l**  *BOLDVariability ~ FramewiseDisplacement + (1 \| SubjectID)* | | | | | | | |
|  | Intercept | 0.39 | 0.03 | t_(97.24)_ = 15.62 | < 0.001*** |  |  |
|  | Framewise Displacement | 0.41 | 0.08 | t_(117.33)_ = 5.29 | < 0.001*** | F_(1,117.33)_ = 28.00 | < 0.001*** |
| **Left A6cdl**  *BOLDVariability_Trans ~ FramewiseDisplacement + TaskCondition * AgeGroup +*  *(1 \| SubjectID)* | | | | | | | |
|  | Intercept | -1.05 | 0.06 | t_(0.01)_ =  -16.58 | < 0.001*** |  |  |
|  | Framewise Displacement | 0.63 | 0.16 | t_(0.01)_ = 3.85 | < 0.001*** | F_(1,128.23)_ = 14.83 | < 0.001*** |
|  | Task Condition [Line 31] | < 0.01 | < 0.01 | t_(0.87)_ = 0.60 | 0.551 | F_(2,88.28)_ = 1.63 | 0.201 |
|  | Task Condition [Angle 31] | < -0.01 | < 0.01 | t_(0.89)_ =  -0.05 | 0.960 |  |  |
|  | Age Group [Younger Adult] | -0.05 | 0.05 | t_(0.58)_ =  -1.01 | 0.316 | F_(1,51.00)_ = 0.87 | 0.360 |
|  | Task Condition [Line 31]:  Age Group [Younger Adult] | -0.02 | 0.02 | t_(0.90)_ =  -0.82 | 0.413 | F_(2,88.94)_ = 3.11 | 0.050 |
|  | Task Condition [Angle 31]:  Age Group [Younger Adult] | 0.04 | 0.02 | t_(0.87)_ = 1.67 | 0.098 |  |  |
| **Left A6dl**  *BOLDVariability_Trans ~ FramewiseDisplacement + TaskCondition + (1 \| SubjectID)* | | | | | | | |
|  | Intercept | -1.70 | 0.07 | t_(85.79)_ =  -22.99 | < 0.001*** |  |  |
|  | Framewise Displacement | 1.31 | 0.24 | t_(98.44)_ = 5.53 | < 0.001*** | F_(1,98.44)_ = 30.59 | < 0.001*** |
|  | Task Condition [Line 31] | 0.02 | 0.02 | t_(83.47)_ = 1.09 | 0.278 | F_(2,84.08)_ = 3.42 | 0.038* |
|  | Task Condition [Angle 31] | 0.05 | 0.02 | t_(85.50)_ = 2.60 | 0.011* |  |  |
| **Right A6dl**  *BOLDVariability_Trans ~ FramewiseDisplacement + AgeGroup + (1 \| SubjectID)* | | | | | | | |
|  | Intercept | -0.86 | 0.05 | t_(96.68)_ =  -17.70 | < 0.001*** |  |  |
|  | Framewise Displacement | 0.56 | 0.13 | t_(118.09)_ = 4.35 | < 0.001*** | F_(1,118.09)_ = 18.94 | < 0.001*** |
|  | Age Group [Younger Adult] | -0.08 | 0.04 | t_(50.68)_ =  -2.27 | 0.028* | F_(1,50.68)_ = 5.16 | 0.028* |
| **Left A6vl**  *BOLDVariability_Trans ~ FramewiseDisplacement + (1 \| SubjectID)* | | | | | | | |
|  | Intercept | -0.99 | 0.06 | t_(104.22)_ =  -16.60 | < 0.001*** |  |  |
|  | Framewise Displacement | 0.79 | 0.18 | t_(127.62)_ = 4.35 | < 0.001*** | F_(1,127.62)_ = 18.89 | < 0.001*** |
| **Right A7c**  *BOLDVariability_Trans ~ FramewiseDisplacement + (1 \| SubjectID)* | | | | | | | |
|  | Intercept | -1.84 | 0.14 | t_(82.65)_ =  -13.69 | < 0.001*** |  |  |
|  | Framewise Displacement | 1.95 | 0.43 | t_(96.24)_ = 4.57 | < 0.001*** | F_(1,96.24)_ = 20.88 | < 0.001*** |
| **Right A7ip**  *BOLDVariability_Trans ~ FramewiseDisplacement + (1 \| SubjectID)* | | | | | | | |
|  | Intercept | -0.98 | 0.06 | t_(101.36)_ =  -17.54 | < 0.001*** |  |  |
|  | Framewise Displacement | 0.89 | 0.17 | t_(122.95)_ = 5.18 | < 0.001*** | F_(1,122.95)_ = 26.78 | < 0.001*** |
| **Right A7m**  *BOLDVariability_Trans ~ FramewiseDisplacement + TaskCondition * AgeGroup +*  *(1 \| SubjectID)* | | | | | | | |
|  | Intercept | -0.90 | 0.07 | t_(96.11)_ =  -13.59 | < 0.001*** |  |  |
|  | Framewise Displacement | 0.77 | 0.17 | t_(113.06)_ = 4.44 | < 0.001*** | F_(1,113.06)_ = 19.69 | < 0.001*** |
|  | Task Condition [Line 31] | 0.03 | 0.02 | t_(89.21)_ = 1.65 | 0.102 | F_(2,90.78)_ = 2.18 | 0.119 |
|  | Task Condition [Angle 31] | 0.02 | 0.02 | t_(91.94)_ = 0.92 | 0.363 |  |  |
|  | Age Group [Younger Adult] | 0.10 | 0.05 | t_(61.25)_ = 1.93 | 0.058 | F_(1,51.27)_ = 3.45 | 0.069 |
|  | Task Condition [Line 31]:  Age Group [Younger Adult] | -0.05 | 0.03 | t_(91.25)_ =  -1.73 | 0.087 | F_(2,91.08)_ = 3.15 | 0.048* |
|  | Task Condition [Angle 31]:  Age Group [Younger Adult] | 0.02 | 0.03 | t_(89.22)_ = 0.76 | 0.448 |  |  |
| **Left A7r**  *BOLDVariability ~ FramewiseDisplacement + TaskCondition + (1 \| SubjectID)* | | | | | | | |
|  | Intercept | 0.35 | 0.02 | t_(86.08)_ = 15.56 | < 0.001*** |  |  |
|  | Framewise Displacement | 0.32 | 0.07 | t_(97.06)_ = 4.44 | < 0.001*** | F_(1,97.06)_ = 19.67 | < 0.001*** |
|  | Task Condition [Line 31] | < 0.01 | < 0.01 | t_(88.56)_ = 0.48 | 0.634 | F_(2,89.36)_ = 4.43 | 0.015* |
|  | Task Condition [Angle 31] | 0.02 | < 0.01 | t_(90.90)_ = 2.78 | 0.007** |  |  |
| **Left iOccG**  *BOLDVariability_Trans ~ FramewiseDisplacement + (1 \| SubjectID)* | | | | | | | |
|  | Intercept | -0.82 | 0.08 | t_(105.97)_ =  -10.14 | < 0.001*** |  |  |
|  | Framewise Displacement | 0.97 | 0.25 | t_(129.63)_ = 3.94 | < 0.001*** | F_(1,129.63)_ =15.54 | < 0.001*** |
| **Left lsOccG**  *BOLDVariability_Trans ~ FramewiseDisplacement + TaskCondition + AgeGroup +*  *(1 \| SubjectID)* | | | | | | | |
|  | Intercept | -5.58 | 0.60 | t_(77.30)_ =  -9.24 | < 0.001*** |  |  |
|  | Framewise Displacement | 6.40 | 1.63 | t_(85.60)_ = 3.92 | < 0.001*** | F_(1,85.60)_ = 15.35 | < 0.001*** |
|  | Task Condition [Line 31] | 0.30 | 0.16 | t_(88.08)_ = 1.90 | 0.061 | F_(2,88.56)_ = 4.74 | 0.011* |
|  | Task Condition [Angle 31] | 0.49 | 0.16 | t_(90.15)_ = 3.05 | 0.003** |  |  |
|  | Age Group [Younger Adult] | -0.95 | 0.42 | t_(48.70)_ =  -2.52 | 0.029* | F_(1,48.70)_ = 5.07 | 0.029* |
| **Left mOccG**  *BOLDVariability_Trans ~ FramewiseDisplacement + AgeGroup + (1 \| SubjectID)* | | | | | | | |
|  | Intercept | -1.25 | 0.11 | t_(99.75)_ =  -11.66 | < 0.001*** |  |  |
|  | Framewise Displacement | 1.25 | 0.28 | t_(122.91)_ = 4.51 | < 0.001*** | F_(1,122.91)_ = 20.37 | < 0.001*** |
|  | Age Group [Younger Adult] | -0.24 | 0.08 | t_(50.90)_ =  -2.85 | < 0.001*** | F_(1,50.90)_ = 8.13 | 0.006** |
| **Left OPC**  *BOLDVariability_Trans ~ FramewiseDisplacement + (1 \| SubjectID)* | | | | | | | |
|  | Intercept | -0.93 | 0.07 | t_(88.19)_ =  -14.13 | < 0.001*** |  |  |
|  | Framewise Displacement | 1.25 | 0.21 | t_(104.46)_ = 6.01 | < 0.001*** | F_(1,104.46)_ = 36.11 | < 0.001*** |
| **Left V5/MT+**  *BOLDVariability_Trans ~ FramewiseDisplacement + TaskCondition * AgeGroup +*  *(1 \| SubjectID)* | | | | | | | |
|  | Intercept | -1.08 | 0.12 | t_(0.01)_ =  -9.13 | < 0.001*** |  |  |
|  | Framewise Displacement | 0.84 | 0.30 | t_(0.03)_ = 2.82 | 0.006** | F_(1,130.02)_ = 7.97 | 0.006** |
|  | Task Condition [Line 31] | 0.01 | 0.03 | t_(0.86)_ = 0.38 | 0.705 | F_(2,86.39)_ = 3.07 | 0.052 |
|  | Task Condition [Angle 31] | < -0.01 | 0.03 | t_(0.87)_ =  -0.04 | 0.972 |  |  |
|  | Age Group [Younger Adult] | -0.10 | 0.10 | t_(0.56)_ =  -0.99 | 0.327 | F_(1,50.01)_ = 0.64 | 0.052 |
|  | Task Condition [Line 31]:  Age Group [Younger Adult] | -0.02 | 0.04 | t_(0.88)_ =  -0.54 | 0.589 | F_(2,87.13)_ = 4.17 | 0.019* |
|  | Task Condition [Angle 31]:  Age Group [Younger Adult] | 0.09 | 0.04 | t_(0.85)_ = 2.22 | 0.029* |  |  |
| **Right V5/MT+**  *BOLDVariability_Trans ~ FramewiseDisplacement + TaskCondition * AgeGroup +*  *(1 \| SubjectID)* | | | | | | | |
|  | Intercept | -1.37 | 0.12 | t_(106.81)_ =  -11.58 | < 0.001*** |  |  |
|  | Framewise Displacement | 1.34 | 0.30 | t_(130.73)_ = 4.53 | < 0.001*** | F_(1,130.73)_ = 20.51 | < 0.001*** |
|  | Task Condition [Line 31] | 0.03 | 0.03 | t_(88.91)_ = 0.99 | 0.325 | F_(2,90.08)_ = 2.47 | 0.090 |
|  | Task Condition [Angle 31] | 0.02 | 0.03 | t_(90.66)_ = 0.66 | 0.510 |  |  |
|  | Age Group [Younger Adult] | 0.05 | 0.10 | t_(58.19)_ = 0.50 | 0.621 | F_(1,51.23)_ = 0.11 | 0.744 |
|  | Task Condition [Line 31]:  Age Group [Younger Adult] | -0.08 | 0.04 | t_(90.55)_ =  -1.91 | 0.059 | F_(2,90.26)_ = 3.53 | 0.034* |
|  | Task Condition [Angle 31]:  Age Group [Younger Adult] | 0.03 | 0.04 | t_(88.92)_ = 0.65 | 0.517 |  |  |
| **Left Crus I**  *BOLDVariability_Trans ~ FramewiseDisplacement + TaskCondition * AgeGroup +*  *(1 \| SubjectID)* | | | | | | | |
|  | Intercept | -0.52 | 0.05 | t_(86.68)_ =  -9.96 | < 0.001*** |  |  |
|  | Framewise Displacement | 0.75 | 0.14 | t_(98.56)_ = 5.41 | < 0.001*** | F_(1,98.56)_ = 39.30 | < 0.001*** |
|  | Task Condition [Line 31] | 0.02 | 0.06 | t_(87.79)_ = 1.32 | 0.190 | F_(2,89.31)_ = 0.18 | 0.840 |
|  | Task Condition [Angle 31] | -0.01 | 0.02 | t_(90.36)_ =  -0.69 | 0.492 |  |  |
|  | Age Group [Younger Adult] | -0.04 | 0.04 | t_(61.83)_ =  -1.08 | 0.283 | F_(1,49.05)_ = 1.27 | 0.265 |
|  | Task Condition  [Line 31]:  Age Group [Younger Adult] | -0.03 | 0.02 | t_(89.84)_ =  -1.34 | 0.182 | F_(2,89.70)_ = 3.98 | 0.02* |
|  | Task Condition [Angle 31]:  Age Group [Younger Adult] | 0.04 | 0.02 | t_(97.90)_ = 1.51 | 0.134 |  |  |
| **Right Crus I**  *BOLDVariability_Trans ~ FramewiseDisplacement + TaskCondition * AgeGroup +*  *(1 \| SubjectID)* | | | | | | | |
|  | Intercept | -0.94 | 0.10 | t_(93.43)_ =  -8.98 | < 0.001*** |  |  |
|  | Framewise Displacement | 1.25 | 0.27 | t_(109.68)_ = 4.58 | < 0.001*** | F_(1,109.68)_ = 20.94 | < 0.001*** |
|  | Task Condition [Line 31] | 0.03 | 0.03 | t_(87.22)_ = 1.03 | 0.305 | F_(2,88.58)_ = 0.07 | 0.935 |
|  | Task Condition [Angle 31] | -0.04 | 0.03 | t_(89.35)_ =  -1.34 | 0.185 |  |  |
|  | Age Group [Younger Adult] | -0.13 | 0.08 | t_(60.15)_ =  -1.54 | 0.128 | F_(1,50.09)_ = 2.14 | 0.150 |
|  | Task Condition [Line 31]:  Age Group [Younger Adult] | -0.05 | 0.04 | t_(89.28)_ =  -1.08 | 0.281 | F_(2,88.92)_ = 4.73 | 0.011* |
|  | Task Condition [Angle 31]:  Age Group [Younger Adult] | 0.08 | 0.04 | t_(87.15)_ = 2.01 | 0.048* |  |  |
| **Left VI**  *BOLDVariability ~ FramewiseDisplacement + (1 \| SubjectID)* | | | | | | | |
|  | Intercept | 0.63 | 0.04 | t_(101.54)_ =  17.57 | < 0.001*** |  |  |
|  | Framewise Displacement | 0.43 | 0,11 | t_(124.01)_ = 3.88 | < 0.001*** | F_(1,124.01)_ = 15.06 | < 0.001*** |
| **Right VI**  *BOLDVariability_Trans ~ FramewiseDisplacement + TaskCondition * AgeGroup +*  *(1 \| SubjectID)* | | | | | | | |
|  | Intercept | -0.62 | 0.07 | t_(101.93)_ =  -8.46 | < 0.001*** |  |  |
|  | Framewise Displacement | 0.59 | 0.19 | t_(122.21)_ = 3.11 | 0.002** | F_(1,122.21)_ = 9.69 | 0.002** |
|  | Task Condition [Line 31] | 0.01 | 0.02 | t_(88.18)_ = 0.54 | 0.589 | F_(2,89.62)_ = 2.76 | 0.069 |
|  | Task Condition [Angle 31] | < 0.01 | 0.02 | t_(90.48)_ = 0.07 | 0.942 |  |  |
|  | Age Group [Younger Adult] | -0.03 | 0.06 | t_(59.54)_ =  -0.42 | 0.679 | F_(1,51.39)_ = 0.11 | 0.745 |
|  | Task Condition [Line 31]:  Age Group [Younger Adult] | -0.03 | 0.03 | t_(90.27)_ =  -1.09 | 0.280 | F_(2,89.95)_ = 3.99 | 0.022* |
|  | Task Condition [Angle 31]:  Age Group [Younger Adult] | 0.05 | 0.03 | t_(88.16)_ = 1.77 | 0.080 |  |  |
| **Left VIIb**  *BOLDVariability_Trans ~ FramewiseDisplacement + (1 \| SubjectID)* | | | | | | | |
|  | Intercept | -0.64 | 0.04 | t_(95.90)_ =  -14.64 | < 0.001*** |  |  |
|  | Framewise Displacement | 0.77 | 0.14 | t_(115.75)_ = 5.67 | < 0.001*** | F_(1,115.75)_ = 32.14 | < 0.001*** |
| **Left VIIIb**  *BOLDVariability_Trans ~ FramewiseDisplacement + AgeGroup + (1 \| SubjectID)* | | | | | | | |
|  | Intercept | -0.02 | 0.06 | t_(104.76)_ =  -0.41 | 0.686 |  |  |
|  | Framewise Displacement | 0.36 | 0.15 | t_(129.03)_ = 2.36 | 0.020* | F_(1,129.03)_ = 5.55 | 0.020* |
|  | Age Group [Younger Adult] | -1.18 | 0.05 | t_(50.64)_ =  -3.68 | < 0.001*** | F_(1,50.64)_ = 13.52 | < 0.001*** |

*Note.* Detailed results from the fixed effects and ANOVA tables derived from the Linear Mixed Models conducted separately for each ROI, examining the relationship between TASK CONDITION, AGE GROUP, their interaction (TASK CONDITION X AGE GROUP), and BOLD Variability (BOLD SD), while including FRAMEWISE DISPLACEMENT as a covariate of no interest and controlling for SUBJECT as a random effect. Model formulas reflect the final statistical model employed for each ROI after stepwise removal of non-significant predictors. Significance levels: * *p* < 0.05, ** *p* < 0.01, *** *p* < 0.001. Abbreviations: ANOVA = Analysis of Variance; $\beta$ = Coefficient Estimate; DenDF = Denominator Degrees of Freedom; NumDF = Numerator Degrees of Freedom; ROI = Region of Interest; SE = Standard Error.
