# Supplementary material for: Age-specific relationship between the modulation of brain dynamics in response to task demands and bimanual performance
Source: Aging (Albany NY). 2026 Mar 24;18(1):159–89. doi: 10.18632/aging.206363 (PMC13285952; doi:10.18632/aging.206363)
Supplement: Supplementary Table 2 [file aging-18-1-206363-s003.docx]

**Supplementary Table 2. Detailed results from linear mixed model with BTT score as dependent variable.**

| Term  [reference level] | | Fixed Effects | | | | ANOVA Type III | |
| --- | --- | --- | --- | --- | --- | --- | --- |
|  |  | $\beta$ | SE | *t* _(df)_ | *p-*value | F-value _(NumDF, DenDF)_ | *p-*value |
| **Right A2**  *BTTscore ~ TaskCondition * BOLDregressed + AgeGroup + BOLDregressed:AgeGroup + (1 \| SubjectID)* | | | | | | | |
|  | Intercept | 40.22 | 7.81 | t_(115.82)_ = 5.15 | < 0.001*** |  |  |
|  | Task Condition [Line 31] | -10.98 | 6.96 | t_(92.50)_  = -1.58 | 0.118 | F_(2,92.00)_ = 5.56 | 0.005** |
|  | Task Condition [Angle 31] | -23.17 | 6.98 | t_(91.94)_  = -3.32 | 0.001** |  |  |
|  | BOLD_REGRESSED_ | -7.44 | 24.71 | t_(121.23)_ = -0.30 | 0.760 | F_(1,95.03)_ = 1.97 | 0.164 |
|  | Age Group [Younger Adult] | 36.68 | 9.70 | t_(88.78)_  = 3.78 | < 0.001*** | F_(1,88.78)_ = 14.31 | < 0.001*** |
|  | Task Condition [Line 31]: BOLD_REGRESSED_ | 30.73 | 23.22 | t_(92.37)_  = 1.32 | 0.189 | F_(2,91.93)_ = 0.90 | 0.412 |
|  | Task Condition [Angle 31]: BOLD_REGRESSED_ | 20.73 | 23.21 | t_(91.97)_  = 0.89 | 0.374 |  |  |
|  | BOLD_REGRESSED_: Age Group [Younger Adult] | -65.23 | 32.60 | t_(94.60)_  = -2.00 | 0.048* | F_(1,94.60)_ = 4.00 | 0.048* |
| **Right A39c**  *BTTscore ~ TaskCondition + AgeGroup + (1 \| SubjectID)* | | | | | | | |
|  | Intercept | 38.13 | 2.12 | t_(66.92)_  = 17.96 | < 0.001*** |  |  |
|  | Task Condition [Line 31] | -1.81 | 1.61 | t_(90.00)_  = -1.13 | 0.262 | F_(2,90)_ = 69.43 | < 0.001*** |
|  | Task Condition [Angle 31] | -17.24 | 1.61 | t_(90.00)_ =  -10.72 | < 0.001*** |  |  |
|  | Age Group [Younger Adult] | 18.49 | 2.73 | t_(45.00)_  = 6.77 | < 0.001*** | F_(1,45)_ = 45.87 | < 0.001*** |
| **Left A40rd**  *BTTscore ~ TaskCondition + BOLDregressed + AgeGroup + BOLDregressed:AgeGroup + (1 \| SubjectID)* | | | | | | | |
|  | Intercept | 28.40 | 5.23 | t_(71.25)_  = 5.44 | < 0.001*** |  |  |
|  | Task Condition [Line 31] | -2.04 | 1.62 | t_(89.14)_  = -1.26 | 0.211 | F_(2,89.41)_ = 66.20 | < 0.001*** |
|  | Task Condition [Angle 31] | -17.05 | 1.62 | t_(89.13)_ =  -10.55 | < 0.001*** |  |  |
|  | BOLD_REGRESSED_ | 31.19 | 15.53 | t_(71.58)_  = 2.01 | 0.048* | F_(1,82.76)_ = 0.21 | 0.649 |
|  | Age Group [Younger Adult] | 41.12 | 9.10 | t_(77.50)_  = 4.52 | < 0.001*** | F_(1,77.50)_ = 20.41 | < 0.001*** |
|  | BOLD_REGRESSED_: Age Group [Younger Adult] | -75.88 | 29.63 | t_(81.82)_  = -2.56 | 0.012* | F_(1,81.82)_ = 6.56 | 0.012* |
| **Left A6dl**  *BTTscore ~ TaskCondition * BOLDregressed + AgeGroup + BOLDregressed:AgeGroup + (1 \| SubjectID)* | | | | | | | |
|  | Intercept | 50.40 | 8.18 | t_(123.78)_ = 6.16 | < 0.001*** |  |  |
|  | Task Condition [Line 31] | -14.13 | 8.26 | t_(92.96)_  = -1.71 | 0.090 | F_(2,92.43)_ = 10.64 | < 0.001*** |
|  | Task Condition [Angle 31] | -37.98 | 8.32 | t_(92.91)_  = -4.56 | < 0.001*** |  |  |
|  | BOLD_REGRESSED_ | -42.63 | 27.18 | t_(127.61)_ = -1.57 | 0.119 | F_(1,102.02)_ = 1.21 | 0.280 |
|  | Age Group [Younger Adult] | 29.54 | 13.03 | t_(96.38)_  = 2.27 | 0.026* | F_(1,96.38)_ = 5.14 | 0.026* |
|  | Task Condition [Line 31]: BOLD_REGRESSED_ | 42.75 | 27.95 | t_(92.90)_  = 1.53 | 0.130 | F_(2,92.66)_ = 3.32 | 0.041* |
|  | Task Condition [Angle 31]: BOLD_REGRESSED_ | 72.14 | 28.14 | t_(93.50)_  = 2.56 | 0.012* |  |  |
|  | BOLD_REGRESSED_: Age Group [Younger Adult] | -38.72 | 43.94 | t_(99.57)_  = -0.88 | 0.380 | F_(1,99.57)_ = 0.78 | 0.380 |
| **Right A6dl**  *BTTscore ~ TaskCondition * BOLDregressed + AgeGroup + (1 \| SubjectID)* | | | | | | | |
|  | Intercept | 50.36 | 6.72 | t_(129.37)_ = 7.49 | < 0.001*** |  |  |
|  | Task Condition [Line 31] | -16.37 | 6.91 | t_(91.81)_  = -2.37 | 0.020* | F_(2,92.16)_ = 15.49 | < 0.001*** |
|  | Task Condition [Angle 31] | -37.74 | 6.81 | t_(92.45)_  = -5.55 | < 0.001*** |  |  |
|  | BOLD_REGRESSED_ | -39.00 | 19.93 | t_(133.80)_ = -1.96 | 0.053 | F_(1,101.47)_ = 0.02 | 0.903 |
|  | Age Group [Younger Adult] | 18.45 | 2.74 | t_(45.79)_  = 6.73 | < 0.001*** | F_(1,45.79)_ = 45.31 | < 0.001*** |
|  | Task Condition [Line 31]: BOLD_REGRESSED_ | 46.32 | 21.25 | t_(91.91)_  = 2.18 | 0.032* | F_(2,92.30)_ = 5.03 | 0.009** |
|  | Task Condition [Angle 31]: BOLD_REGRESSED_ | 65.03 | 21.01 | t_(92.69)_  = 3.10 | 0.003** |  |  |
| **Right A7m**  *BTTscore ~ TaskCondition + BOLDregressed + AgeGroup + (1 \| SubjectID)* | | | | | | | |
|  | Intercept | 26.98 | 5.33 | t_(105.99)_ = 5.06 | < 0.001*** |  |  |
|  | Task Condition [Line 31] | -2.18 | 1.57 | t_(89.83)_  = -1.39 | 0.167 | F_(2,89.68)_ = 76.05 | < 0.001*** |
|  | Task Condition [Angle 31] | -17.70 | 1.57 | t_(90.40)_ =  -11.27 | < 0.001*** |  |  |
|  | BOLD_REGRESSED_ | 26.58 | 11.67 | t_(114.54)_ = 2.28 | 0.025* | F_(1,114.54)_ = 5.20 | 0.025* |
|  | Age Group [Younger Adult] | 17.51 | 2.79 | t_(44.82)_  = 6.29 | < 0.001*** | F_(1,44.82)_ = 39.50 | < 0.001*** |
| **Left A7r**  *BTTscore ~ TaskCondition + AgeGroup + (1 \| SubjectID)* | | | | | | | |
|  | Intercept | 38.13 | 2.12 | t_(66.92)_  = 17.96 | < 0.001*** |  |  |
|  | Task Condition [Line 31] | -1.81 | 1.61 | t_(90.00)_  = -1.13 | 0.062 | F_(2,90)_ = 69.43 | < 0.001*** |
|  | Task Condition [Angle 31] | -17.24 | 1.61 | t_(90.00)_ =  -10.72 | < 0.001*** |  |  |
|  | Age Group [Younger Adult] | 18.49 | 2.73 | t_(45.00)_  = 6.77 | < 0.001*** | F_(1,45)_ = 45.87 | < 0.001*** |
| **Left lsOccG**  *BTTscore ~ TaskCondition + BOLDregressed + AgeGroup + (1 \| SubjectID)* | | | | | | | |
|  | Intercept | 33.17 | 2.93 | t_(92.49)_  = 11.32 | < 0.001*** |  |  |
|  | Task Condition [Line 31] | -2.27 | 1.58 | t_(91.12)_  = -1.44 | 0.155 | F_(2,90.49)_ = 72.87 | < 0.001*** |
|  | Task Condition [Angle 31] | -17.44 | 1.57 | t_(90.10)_=  -11.09 | < 0.001*** |  |  |
|  | BOLD_REGRESSED_ | 19.49 | 8.09 | t_(121.60)_ = 2.41 | 0.018* | F_(1,121.60)_ = 5.81 | 0.018* |
|  | Age Group [Younger Adult] | 18.82 | 2.69 | t_(44.97)_  = 7.00 | < 0.001*** | F_(1,44.97)_ = 48.99 | < 0.001*** |
| **Left mOccG**  *BTTscore8 ~ TaskCondition * BOLDregressed + AgeGroup + (1 \| SubjectID)* | | | | | | | |
|  | Intercept | 43.91 | 3.97 | t_(130.94)_ = 11.06 | < 0.001*** |  |  |
|  | Task Condition [Line 31] | -13.58 | 4.62 | t_(97.88)_  = -2.94 | 0.004** | F_(2,95.91)_ = 10.61 | < 0.001*** |
|  | Task Condition [Angle 31] | -21.44 | 4.85 | t_(97.88)_  = -4.42 | < 0.001*** |  |  |
|  | BOLD_REGRESSED_ | -18.67 | 10.38 | t_(129.44)_ = -1.80 | 0.074 | F_(1,131.18)_ = 0.01 | 0.907 |
|  | Age Group [Younger Adult] | 18.42 | 2.79 | t_(46.39)_  = 6.60 | < 0.001*** | F_(1,46.39)_ = 43.62 | < 0.001*** |
|  | Task Condition [Line 31]: BOLD_REGRESSED_ | 38.88 | 14.51 | t_(97.59)_  = 2.68 | 0.009** | F_(2,96.84)_ = 3.60 | 0.031* |
|  | Task Condition [Angle 31]: BOLD_REGRESSED_ | 13.72 | 14.86 | t_(98.65)_  = 0.92 | 0.358 |  |  |
| **Left V5/MT+**  *BTTscore ~ TaskCondition * BOLDregressed + AgeGroup + (1 \| SubjectID)* | | | | | | | |
|  | Intercept | 42.90 | 4.88 | t_(117.30)_ = 8.79 | < 0.001*** |  |  |
|  | Task Condition [Line 31] | -15.07 | 4.93 | t_(91.69)_  = -3.06 | 0.003** | F_(2,91.62)_ = 11.77 | < 0.001*** |
|  | Task Condition [Angle 31] | -23.10 | 4.83 | t_(91.82)_  = -4.78 | < 0.001*** |  |  |
|  | BOLD_REGRESSED_ | -13.31 | 11.96 | t_(128.25)_ = -1.11 | 0.268 | F_(1,94.61)_ = 0.19 | 0.662 |
|  | Age Group [Younger Adult] | 18.63 | 2.74 | t_(45.12)_  = 6.81 | < 0.001*** | F_(1,45.12)_ = 46.37 | < 0.001*** |
|  | Task Condition [Line 31]: BOLD_REGRESSED_ | 36.57 | 12.90 | t_(45.12)_  = 2.84 | 0.006** | F_(2,91.96)_ = 4.03 | 0.021* |
|  | Task Condition [Angle 31]: BOLD_REGRESSED_ | 16.06 | 12.42 | t_(92.07)_  = 1.29 | 0.199 |  |  |
| **Right V5/MT+**  *BTTscore ~ TaskCondition + AgeGroup + (1 \| SubjectID)* | | | | | | | |
|  | Intercept | 38.13 | 2.12 | t_(66.92)_  = 17.96 | < 0.001*** |  |  |
|  | Task Condition [Line 31] | -1.81 | 1.61 | t_(90.00)_  = -1.13 | 0.262 | F_(2,90)_ = 69.43 | < 0.001*** |
|  | Task Condition [Angle 31] | -17.24 | 1.61 | t_(90.00)_ =  -10.72 | < 0.001*** |  |  |
|  | Age Group [Younger Adult] | 18.49 | 2.73 | t_(45.00)_  = 6.77 | < 0.001*** | F_(1,45)_ = 45.87 | < 0.001*** |
| **Left Crus I**  *BTTscore ~ TaskCondition * BOLDregressed + AgeGroup + BOLDregressed:AgeGroup + (1 \| SubjectID)* | | | | | | | |
|  | Intercept | 49.96 | 7.28 | t_(122.28)_ = 6.86 | < 0.001*** |  |  |
|  | Task Condition [Line 31] | -21.47 | 6.35 | t_(96.00)_  = -3.38 | 0.001** | F_(2,93.87)_ =  12.25 | < 0.001*** |
|  | Task Condition [Angle 31] | -32.85 | 6.69 | t_(92.37)_  = -4.91 | < 0.001*** |  |  |
|  | BOLD_REGRESSED_ | -25.24 | 14.66 | t_(126.99)_ = -1.72 | 0.088 | F_(1,102.19)_ = 4.40 | 0.038* |
|  | Age Group [Younger Adult] | 38.80 | 10.19 | t_(95.56)_  = 3.81 | < 0.001*** | F_(1,95.56)_ = 14.49 | < 0.001*** |
|  | Task Condition [Line 31]: BOLD_REGRESSED_ | 40.76 | 13.01 | t_(97.30)_  = 3.13 | 0.002** | F_(2,94.48)_ = 5.16 | 0.008** |
|  | Task Condition [Angle 31]: BOLD_REGRESSED_ | 33.69 | 13.78 | t_(92.33)_  = 2.44 | 0.016* |  |  |
|  | BOLD_REGRESSED_: Age Group [Younger Adult] | -43.34 | 20.94 | t_(100.71)_ = -2.07 | 0.041* | F_(1,100.71)_ = 4.28 | 0.041* |
| **Right Crus I**  *BTTscore ~ TaskCondition + AgeGroup + (1 \| SubjectID)* | | | | | | | |
|  | Intercept | 38.13 | 2.12 | t_(66.92)_  = 17.96 | < 0.001*** |  |  |
|  | Task Condition [Line 31] | -1.81 | 1.61 | t_(90.00)_  = -1.13 | 0.262 | F_(2,90.00)_ = 69.43 | < 0.001*** |
|  | Task Condition [Angle 31] | -17.24 | 1.61 | t_(90.00)_ =  -10.72 | < 0.001*** |  |  |
|  | Age Group [Younger Adult] | 18.49 | 2.73 | t_(45.00)_  = 6.77 | < 0.001*** | F_(1,45.00)_ = 45.87 | < 0.001*** |
| **Right VI**  *BTTscore ~ TaskCondition + AgeGroup + (1 \| SubjectID)* | | | | | | | |
|  | Intercept | 38.13 | 2.12 | t_(66.92)_  = 17.96 | < 0.001*** |  |  |
|  | Task Condition [Line 31] | -1.81 | 1.61 | t_(90.00)_  = -1.13 | 0.262 | F_(2,90.00)_ = 69.43 | < 0.001*** |
|  | Task Condition [Angle 31] | -17.24 | 1.61 | t_(90.00)_ =  -10.72 | < 0.001*** |  |  |
|  | Age Group [Younger Adult] | 18.49 | 2.73 | t_(45.00)_  = 6.77 | < 0.001*** | F_(1,45.00)_ = 45.87 | < 0.001*** |
| **Left VIIb**  *BTTscore ~ TaskCondition + AgeGroup + (1 \| SubjectID)* | | | | | | | |
|  | Intercept | 38.13 | 2.12 | t_(66.92)_ = 17.96 | < 0.001*** |  |  |
|  | Task Condition [Line 31] | -1.81 | 1.61 | t_(90.00)_  = -1.13 | 0.262 | F_(2,90.00)_ = 69.43 | < 0.001*** |
|  | Task Condition [Angle 31] | -17.24 | 1.61 | t_(90.00)_ =  -10.72 | < 0.001*** |  |  |
|  | Age Group [Younger Adult] | 18.49 | 2.73 | t_(45.00)_  = 6.77 | < 0.001*** | F_(1,45.00)_ = 45.87 | < 0.001*** |
| **Left VIIIb**  *BTTscore ~ TaskCondition * BOLDregressed + AgeGroup + BOLDregressed:AgeGroup + (1 \| SubjectID)* | | | | | | | |
|  | Intercept | 44.17 | 6.83 | t_(120.28)_ = 6.46 | < 0.001*** |  |  |
|  | Task Condition [Line 31] | -16.59 | 5.95 | t_(91.50)_  = -2.79 | 0.007** | F_(2,91.85)_ = 11.74 | < 0.001*** |
|  | Task Condition [Angle 31] | -28.94 | 5.98 | t_(92.23)_  = -4.84 | < 0.001*** |  |  |
|  | BOLD_REGRESSED_ | -8.60 | 8.37 | t_(128.16)_ = -1.03 | 0.306 | F_(1,85.96)_ = 0.13 | 0.718 |
|  | Age Group [Younger Adult] | 27.59 | 11.73 | t_(82.53)_  = 2.35 | 0.021* | F_(1,82.53)_ = 5.53 | 0.021* |
|  | Task Condition [Line 31]: BOLD_REGRESSED_ | 20.12 | 7.86 | t_(91.47)_  = 2.35 | 0.012* | F_(2,91.77)_ = 3.59 | 0.032* |
|  | Task Condition [Angle 31]: BOLD_REGRESSED_ | 16.13 | 7.86 | t_(92.47)_ = 2.05 | 0.043* |  |  |
|  | BOLD_REGRESSED_: Age Group [Younger Adult] | -12.95 | 16.53 | t_(85.03)_  = -0.78 | 0.435 | F_(1,85.03)_ = 0.61 | 0.435 |

*Note.* Detailed results from the fixed effects and ANOVA tables derived from the Linear Mixed Models conducted separately for each ROI, examining the relationship between TASK CONDITION, BOLD_REGRESSED_ (BOLD SD after regressing out framewise displacement), AGE GROUP, the two-way interactions of BOLD_REGRESSED_ with the two other predictors (TASK CONDITION X BOLD_REGRESSED_ and AGE GROUP X BOLD_REGRESSED_), and BTT SCORE, while controlling for SUBJECT as a random effect. Model formulas reflect the final statistical model employed for each ROI after stepwise removal of non-significant predictors. Significance levels: * *p* < 0.05, ** *p* < 0.01, *** *p* < 0.001. Abbreviations: ANOVA = Analysis of Variance; $\beta$ = Coefficient Estimate; DenDF = Denominator Degrees of Freedom; NumDF = Numerator Degrees of Freedom; ROI = Region of Interest; SE = Standard Error.
