# Supplementary material for: Age-specific relationship between the modulation of brain dynamics in response to task demands and bimanual performance
Source: Aging (Albany NY). 2026 Mar 24;18(1):159–89. doi: 10.18632/aging.206363 (PMC13285952; doi:10.18632/aging.206363)
Supplement: Supplementary Table 3 [file aging-18-1-206363-s004.docx]

**Supplementary Table 3. Detailed results from multiple linear regression with mean BTT score as dependent variable.**

| Term  [reference level] | | Coefficients | | | | ANOVA Type III | |
| --- | --- | --- | --- | --- | --- | --- | --- |
|  |  | $\beta$ | SE | *t* | *p-*value | F-value _(DF)_ | *p-*value |
| **Right A2**  *MeanBTTScore ~ ModulationBoldSD * AgeGroup* | | | | | | | |
|  | Intercept | 25.68 | 3.74 | 6.87 | < 0.001*** | F_(1)_ = 47.14 | < 0.001*** |
|  | Modulation  BOLD SD | 52.01 | 30.95 | 1.68 | 0.095 | F_(1)_ = 2.83 | 0.095 |
|  | Age Group [Younger Adult] | 38.57 | 5.79 | 6.66 | < 0.001*** | F_(1)_ = 44.36 | < 0.001*** |
|  | Modulation  BOLD SD:  Age Group [Younger Adult] | -215.52 | 58.71 | -3.67 | < 0.001*** | F_(1)_ = 13.47 | < 0.001*** |
| **Right A39c**  *MeanBTTScore ~ AgeGroup* | | | | | | | |
|  | Intercept | 31.68 | 1.15 | 37.61 | < 0.001*** | F_(1)_ = 762.12 | < 0.001*** |
|  | Age Group [Younger Adult] | 18.59 | 1.62 | 11.45 | < 0.001*** | F_(1)_ = 131.19 | < 0.001*** |
| **Left A40rd**  *MeanBTTScore ~ ModulationBoldSD * AgeGroup* | | | | | | | |
|  | Intercept | 20.09 | 2.88 | 6.97 | < 0.001*** | F_(1)_ = 48.61 | < 0.001*** |
|  | Modulation  BOLD SD | 106.07 | 24.43 | 4.34 | < 0.001*** | F_(1)_ = 18.85 | < 0.001*** |
|  | Age Group [Younger Adult] | 31.68 | 3.75 | 8.46 | < 0.001*** | F_(1)_ = 71.57 | < 0.001*** |
|  | Modulation  BOLD SD:  Age Group [Younger Adult] | -123.33 | 34.74 | -3.55 | < 0.001*** | F_(1)_ = 12.60 | < 0.001*** |
| **Left A6dl**  *MeanBTTScore ~ AgeGroup* | | | | | | | |
|  | Intercept | 31.68 | 1.15 | 17.61 | < 0.001*** | F_(1)_ = 762.12 | < 0.001*** |
|  | Age Group [Younger Adult] | 18.59 | 1.62 | 11.45 | < 0.001*** | F_(1)_ = 131.19 | < 0.001*** |
| **Right A6dl**  *MeanBTTScore ~ AgeGroup* | | | | | | | |
|  | Intercept | 31.94 | 1.19 | 26.55 | < 0.001*** | F_(1)_ = 705.19 | < 0.001*** |
|  | Age Group [Younger Adult] | 18.79 | 1.66 | 11.34 | < 0.001*** | F_(1)_ = 128.53 | < 0.001*** |
| **Right A7m**  *MeanBTTScore ~ ModulationBoldSD * AgeGroup* | | | | | | | |
|  | Intercept | 26.65 | 3.39 | 7.86 | < 0.001*** | F_(1)_ = 61.83 | < 0.001*** |
|  | Modulation  BOLD SD | 41.16 | 26.10 | 1.58 | 0.117 | F_(1)_ = 2.49 | 0.117 |
|  | Age Group [Younger Adult] | 29.22 | 4.64 | 6.30 | < 0.001*** | F_(1)_ = 39.73 | < 0.001*** |
|  | Modulation  BOLD SD:  Age Group [Younger Adult] | -81.95 | 33.85 | -2.42 | 0.017* | F_(1)_ = 5.86 | 0.017* |
| **Left A7r**  *MeanBTTScore ~ ModulationBoldSD * AgeGroup* | | | | | | | |
|  | Intercept | 22.33 | 2.54 | 8.78 | < 0.001*** | F_(1)_ = 77.14 | < 0.001*** |
|  | Modulation  BOLD SD | 87.12 | 21.50 | 4.05 | < 0.001*** | F_(1)_ = 16.42 | < 0.001*** |
|  | Age Group [Younger Adult] | 36.35 | 4.46 | 8.15 | < 0.001*** | F_(1)_ = 66.43 | < 0.001*** |
|  | Modulation  BOLD SD:  Age Group [Younger Adult] | -172.45 | 41.55 | -4.15 | < 0.001*** | F_(1)_ = 17.22 | < 0.001*** |
| **Left lsOccG**  *MeanBTTScore ~ ModulationBoldSD * AgeGroup* | | | | | | | |
|  | Intercept | 25.63 | 2.74 | 9.35 | < 0.001*** | F_(1)_ = 87.42 | < 0.001*** |
|  | Modulation  BOLD SD | 51.28 | 21.16 | 2.42 | 0.017* | F_(1)_ = 5.87 | 0.017* |
|  | Age Group [Younger Adult] | 24.12 | 4.80 | 5.02 | < 0.001*** | F_(1)_ = 25.20 | < 0.001*** |
|  | Modulation  BOLD SD:  Age Group [Younger Adult] | -45.55 | 46.55 | -0.98 | 0.330 | F_(1)_ = 0.96 | 0.330 |
| **Left mOccG**  *MeanBTTScore ~ ModulationBoldSD + AgeGroup* | | | | | | | |
|  | Intercept | 27.95 | 1.83 | 15.31 | < 0.001*** | F_(1)_ = 234.39 | < 0.001*** |
|  | Modulation  BOLD SD | 25.66 | 9.88 | 2.60 | 0.011* | F_(1)_ = 6.74 | 0.011* |
|  | Age Group [Younger Adult] | 19.82 | 1.66 | 11.96 | < 0.001*** | F_(1)_ = 143.00 | < 0.001*** |
| **Left V5/MT+**  *MeanBTTScore ~ ModulationBoldSD * AgeGroup* | | | | | | | |
|  | Intercept | 26.81 | 2.25 | 11.90 | < 0.001*** | F_(1)_ = 141.69 | < 0.001*** |
|  | Modulation  BOLD SD | 36.44 | 14.83 | 2.46 | 0.015* | F_(1)_ = 6.04 | 0.015* |
|  | Age Group [Younger Adult] | 36.92 | 4.10 | 9.00 | < 0.001*** | F_(1)_ = 81.15 | < 0.001*** |
|  | Modulation  BOLD SD:  Age Group [Younger Adult] | -156.75 | 32.67 | -4.80 | < 0.001*** | F_(1)_ = 23.02 | < 0.001*** |
| **Right V5/MT+**  *MeanBTTScore ~ ModulationBoldSD * AgeGroup* | | | | | | | |
|  | Intercept | 23.84 | 3.26 | 7.31 | < 0.001*** | F_(1)_ = 53.43 | < 0.001*** |
|  | Modulation  BOLD SD | 62.14 | 24.37 | 2.55 | 0.012* | F_(1)_ = 6.50 | 0.012* |
|  | Age Group [Younger Adult] | 37.79 | 4.84 | 7.80 | < 0.001*** | F_(1)_ = 60.89 | < 0.001*** |
|  | Modulation  BOLD SD:  Age Group [Younger Adult] | -168.31 | 40.14 | -4.19 | < 0.001*** | F_(1)_ = 17.58 | < 0.001*** |
| **Left Crus I**  *MeanBTTScore ~ ModulationBoldSD + AgeGroup* | | | | | | | |
|  | Intercept | 39.08 | 2.66 | 14.68 | < 0.001*** | F_(1)_ = 215.45 | < 0.001*** |
|  | Modulation  BOLD SD | -38.18 | 12.48 | -3.06 | 0.003** | F_(1)_ = 9.35 | 0.003** |
|  | Age Group [Younger Adult] | 17.22 | 1.64 | 10.52 | < 0.001*** | F_(1)_ = 110.74 | < 0.001*** |
| **Right Crus I**  *MeanBTTScore ~ AgeGroup* | | | | | | | |
|  | Intercept | 31.68 | 1.15 | 27.61 | < 0.001*** | F_(1)_ = 762.12 | < 0.001*** |
|  | Age Group [Younger Adult] | 18.59 | 1.62 | 11.45 | < 0.001*** | F_(1)_ = 131.19 | < 0.001*** |
| **Right VI**  *MeanBTTScore ~ ModulationBoldSD * AgeGroup* | | | | | | | |
|  | Intercept | 27.39 | 4.15 | 6.65 | < 0.001*** | F_(1)_ = 44.20 | < 0.001*** |
|  | Modulation  BOLD SD | 28.61 | 26.48 | 1.08 | 0.282 | F_(1)_ = 1.17 | 0.282 |
|  | Age Group [Younger Adult] | 45.09 | 7.82 | 5.77 | < 0.001*** | F_(1)_ = 33.23 | < 0.001*** |
|  | Modulation  BOLD SD:  Age Group [Younger Adult] | -191.81 | 54.97 | -3.49 | < 0.001*** | F_(1)_ = 12.17 | < 0.001*** |
| **Left VIIb**  *MeanBTTScore ~ ModulationBoldSD + AgeGroup* | | | | | | | |
|  | Intercept | 26.70 | 3.18 | 8.39 | < 0.001*** | F_(1)_ = 70.40 | < 0.001*** |
|  | Modulation  BOLD SD | 28.22 | 16.57 | 1.70 | 0.091 | F_(1)_ = 2.90 | 0.091 |
|  | Age Group [Younger Adult] | 19.94 | 1.79 | 11.13 | < 0.001*** | F_(1)_ = 123.87 | < 0.001*** |
| **Left VIIIb**  *MeanBTTScore ~ AgeGroup* | | | | | | | |
|  | Intercept | 31.60 | 1.15 | 27.58 | < 0.001*** | F_(1)_ = 760.44 | < 0.001*** |
|  | Age Group [Younger Adult] | 18.68 | 1.62 | 11.53 | < 0.001*** | F_(1)_ = 132.90 | < 0.001*** |

*Note.* Detailed results from the coefficient and ANOVA tables derived from the Multiple Linear Regressions conducted for each ROI, examining the relationship between AGE GROUP, BOLD SD MODULATION, their interaction, and MEAN BTT SCORE. Model formulas reflect the final statistical model employed for each ROI after stepwise removal of non-significant predictors. Significance levels: * *p* < 0.05, ** *p* < 0.01, *** *p* < 0.001. Abbreviations: ANOVA = Analysis of Variance; $\beta$ = Coefficient Estimate; DF = Degrees of Freedom; ROI = Region of Interest; SE = Standard Error.
